# Nanoarchitectonics of molecular machines, biomolecular machines, and microrobots in their collective behaviour

**DOI:** 10.1039/d6sc01674j

**Published:** 2026-04-29

**Authors:** Katsuhiko Ariga, Wenyan Lyu, Jingwen Song

**Affiliations:** a Research Center for Materials Nanoarchitectonics, National Institute for Materials Science (NIMS) 1-1 Namiki Tsukuba 305-0044 Japan ARIGA.Katsuhiko@nims.go.jp; b Graduate School of Frontier Sciences, The University of Tokyo 5-1-5 Kashiwa-no-ha Kashiwa 277-8561 Japan; c Key Laboratory of Multifunctional Nanomaterials and Smart Systems, Suzhou Institute of Nano-tech and Nano-bionics (SINANO), Chinese Academy of Sciences (CAS) Suzhou 215123 P. R. China

## Abstract

Nanoarchitectonics of small-scale molecular units into functional materials is a key strategy in materials science. The central challenge lies in assembling, integrating, and nanoarchitecting fundamental functional units, such as molecular machines and microrobots, to innovate materials. This review examines collective behaviours typical in molecular and biomolecular machines and microrobots. We categorize collective behaviours into six types: (i) small-scale units operating cooperatively or collaboratively (often seen in macroscopic objects such as crystals); (ii) free-moving units in the bulk material; (iii) mechanical coupling between units resulting in chain interactions (not unlike gears); (iv) assembly of units to generate macroscopic output functions; (v) macroscopic stimuli controlling individual units; and (vi) cooperation between different molecular species to generate functional flows within a single system. After categorizing collective behaviours in molecular machines, we consider examples of material systems exhibiting this behaviour, such as MOFs, COFs and crystals. Furthermore, we examine the collective behaviours of molecular machines at solid surfaces and liquid interfaces. Additionally, we highlight the behaviour of biomolecular machines and microrobots in separate sections. Each section identifies and discusses trends in the relevant examples. This approach to utilizing molecular machines is anticipated to create highly functional systems, realizing the ultimate goal of functional materials chemistry.

## Abstract 2

This review discusses the future of molecular machines and microrobots, focusing on the shift from individual to collective behavior. It also examines how this collective property can be utilized in functionally organized materials.

## Graphical abstract

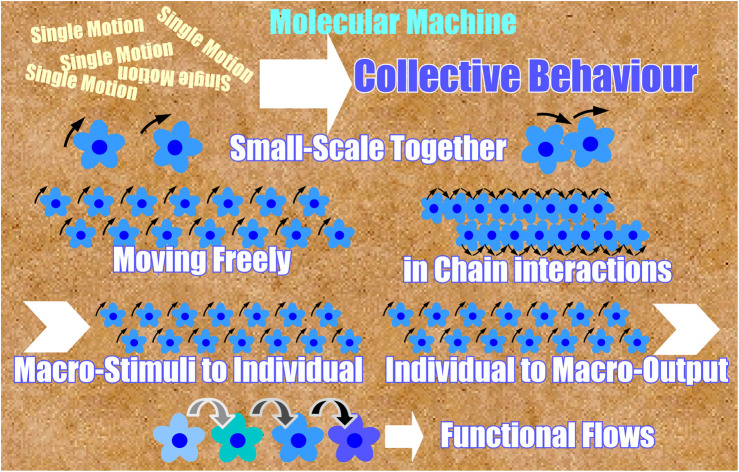

## Introduction

Throughout the history of science, the development of materials has preceded recognition of the significance of structural control. Chemical sciences have significantly expanded the available material repertoire.^1^ The emergence of nanotechnology and nanoscience^2^ further transformed our ability to observe^3^ and manipulate^4^ structures at the atomic and molecular level, unveiling still more unique properties of nanoscale materials and the physics of nanospaces.^5^ Among various nanoobjects, molecular machines are currently the pinnacle of research aiming to create smaller, more precisely architected functional structures.^6^ They are defined as discrete molecules or assemblies of components designed to perform mechanical, directional movements in response to specific energy inputs. Parallelly, nanobots and microrobots are another category of microscopic objects made of nanoparticles or micromaterials that exhibit spontaneous propulsive motion.^7^

Biomolecules are great bases for micromachines and microrobots, as demonstrated in DNA manipulation technology.^8^ In fact, biological processes often involve instances of microscale mechanical movement driven by biomolecular motors,^9^ and the implicated molecules can therefore be considered biomolecular machines. If we extend the definition of mechanical manipulation to include processes such as shape transformation, then enzymes^10^ and membrane channels^11^ are also biomolecular machines.

**Fig. 1:**
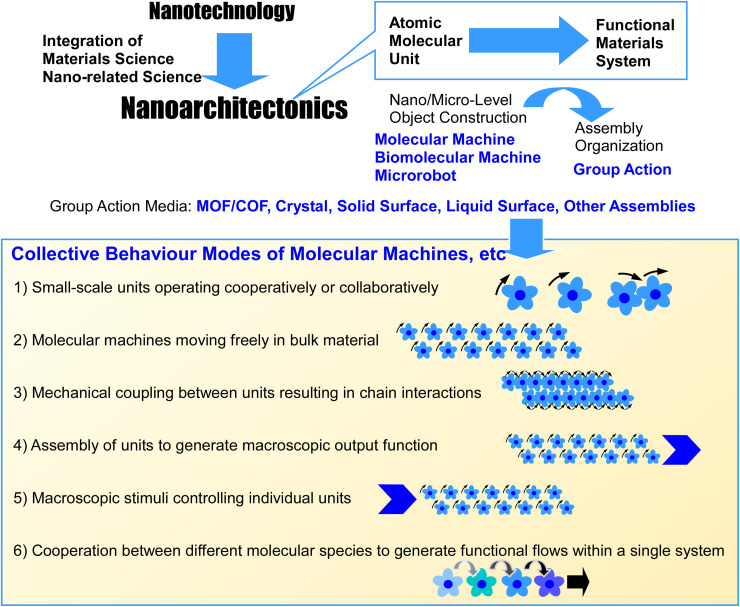
Outline of the nanoarchitectonics concept (top) and the modes of collective behaviour in molecular machines.

In short, the aim is to develop functional machine-like objects from molecules, nano- and micro-materials, and biomolecules. The current challenge lies in assembling and integrating molecular machines and microrobots to perform complex functions in a compact form factor. Real life problems require coordination of compartmentalized functions to be solved. This is most obvious in biological systems, which comprise multiple functional units,^12^ such as biomolecular machines, working in a coordinated manner. This quintessentially demonstrates how smaller components can collaboratively yield highly functional macroscopic outputs. This coincides with the concept of nanoarchitectonics (Fig. 1),^13^ which is described below.

Nanoarchitectonics is defined as building functional materials constructively from fundamental units,^14^ such as atoms, molecules and nano-objects.^15^ Techniques from various fields, including atomic and molecular manipulation, chemical transformation (such as organic synthesis), physical material transformation, spontaneous processes (such as self-assembly and self-organization), orientation and organization control using external forces and fields, nanofabrication, microfabrication and biochemical processes can be combined to transform and organize the fundamental units.^16^ This approach creates functional materials by leveraging the properties of nanostructures, so its application is not limited to specific materials or applications. In a sense, since all matter is made up of atoms and molecules, the nanoarchitectonics methodology can be applied to all matter. If the ultimate theory of physics is the theory of everything,^17^ then nanoarchitectonics could be considered a method for everything in materials science.^18^

Applying the concept of nanoarchitectonics could be a productive approach to developing molecular machines and microrobots. Armed with a large and varied repertoire of nanoscale materials, as well as techniques to manipulate and observe them at the molecular level, we are well equipped to take on the next challenge in assembling and combining these units to create functional materials.

With the ultimate aim of creating artificial systems comprising coordinated functional units yielding remarkable functions in mind, this review discusses the nanoarchitectonics of molecular machines, biomolecular machines and microrobots. It must be noted that this topic entails many unsolved challenges and technologies still in their infancy, so the collective behaviours involving the described machines may not all perfectly align with our prescribed categorization. Nevertheless, we crudely envision the following categories of collective behaviours: (i) units working in tandem, such as gear-like intertwining of molecular rotors, as observed in crystalline lattices; (ii) machines moving freely within a bulk material or substance; (iii) ensembles of interacting molecular components generating interconnected, gear-like motions; (iv) collective action of molecular machines to generate a unified macroscopic output; (v) macroscopic stimuli governing the behaviour of individual units within an assembly; and (vi) diverse machine types working within a single system to produce functional flows.

Given the diversity of these behavioural patterns, this review organizes examples by material, system and environment, rather than by behavioural pattern alone. Nevertheless, we do examine potential associations between materials and systems and corresponding behavioural patterns. We first delve into the categories of collective behaviours we have identified, and then we exemplify molecular machines exhibiting such behaviours in material systems such as MOFs, COFs, and crystals. Next, we examine collective behaviours of molecular machines on solid surfaces and in liquid interfacial environments. Separate sections are dedicated to the distinct behaviours of biomolecular machines and microrobots. While the examples presented are not exhaustive, they demonstrate the types of collective behaviours that can be observed in molecular machines and microrobots in various materials and environments.

The aim of this review is to point out the way forward for cutting-edge objects such as molecular machines and microrobots. Until now, researchers have focused on the individual operation of molecular machines. However, the path to future functional materials lies with exploring collective behaviour. As the research is gaining momentum, this review will examine trends across material systems and environments. We explore the paradigm shift from individual to collective behaviour, while contemplating how this property can be applied in functionally organized materials.

## Some examples of molecular machine collective behaviour in assemblies

**Fig. 2:**
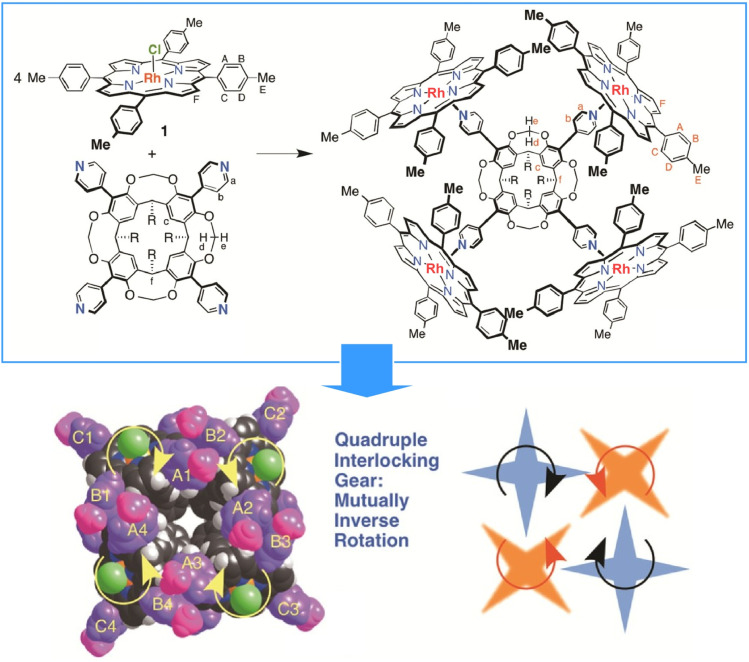
A molecular gear operating within a complex self-assembled from a 4 : 1 complex of Rh(iii)Cl tetrakis(4-methylphenyl)porphyrin and the bowl-shaped tetra(4-pyridyl)cavitand *via* axial coordination bonds between the Rh-pyridyl groups. Reprinted with permission from ref. 19, Copyright 2016 American Chemical Society.

In this section, we will showcase the collective action of molecular machines within molecular and ensemble systems. Fig. 2 shows a molecular gear operating within a complex molecule as described by Kobori, Kobayashi and co-workers.^19^ This gear self-assembles from a 4 : 1 complex of Rh(iii)Cl tetrakis(4-methylphenyl)porphyrin and the bowl-shaped tetra(4-pyridyl)cavitand *via* axial coordination bonds between the Rh-pyridyl groups. This self-assembled complex behaves as a quadruple interlocking gear with an internal space. This molecular gear system comprises four connected gears. The extremely strong porphyrin-Rh-pyridyl axial coordination bond, the rigidity of the methylene-bridged cavitand acting as a scaffold for the pyridyl axis, and the cruciform arrangement of interlocking *p*-tolyl groups acting as the teeth enable this molecular assembly to function as a 14-unit gear. The cross-shaped arrangement of the four aryl groups acting as gear teeth allows the molecule to function as a quadruple interlocking gear in solution without a sliding or rotational mechanism. This example demonstrates how the clever design of molecular components can enable collective behaviours, whereby the molecules work together like gears.

Another example of coordinated molecular motion in an assembly is the supramolecular gear. D'Agostino, Chierotti, Katrusiak and co-workers investigated the structure and solid-state dynamics of the supramolecular salt, (12-crown-4)_2_·DABCO·H_2_O·(X)_2_ (where X = BF_4_ or ClO_4_, and DABCO = 1,4-diazabicyclo[2.2.2]octane).^20^ All components of the supramolecular complex demonstrate dynamic processes at room temperature and pressure. The crown ether motion is maintained even at lower temperatures and higher pressures. This is because neighbouring molecules mesh and rotate together like helical gears. Pressure can trigger the engagement and disengagement of these gears. By optimizing the molecular design and assembly structure, artificial molecular rotors can exhibit coordinated collective behaviours.

**Fig. 3:**
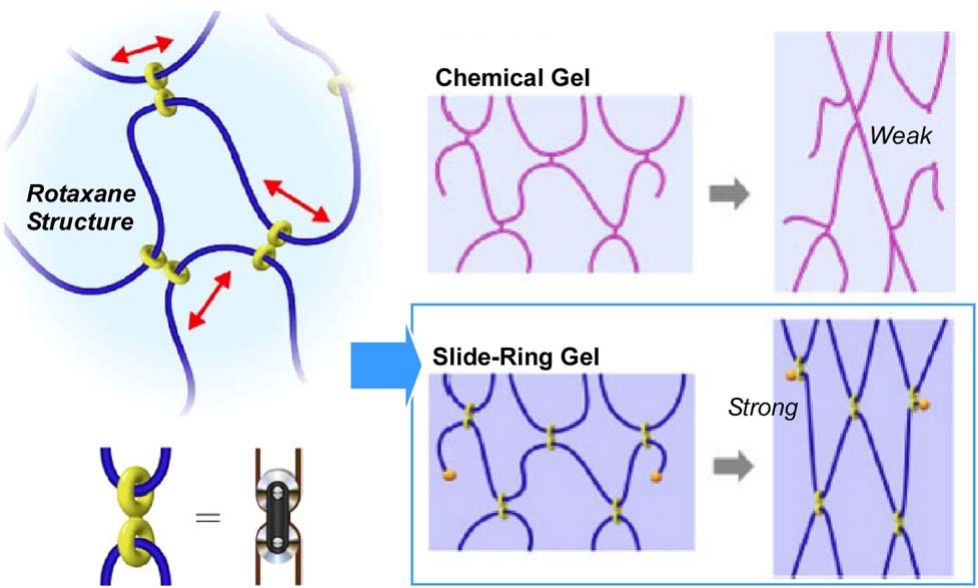
Supramolecular gel structure known as a slide-ring gel boasting topological properties derived from its rotaxane structure, in which polymer chains with bulky end groups are topologically connected by figure-eight crosslinks rather than covalent crosslinks (chemical gels) or attractive interactions (physical gels). Reprinted with permission from ref. 21, Copyright 2007 Springer-Nature.

Some materials are designed to exhibit excellent properties through the cooperative action of molecular machines, even without direct contact. The research group led by Ito *et al.* developed a supramolecular gel structure boasting topological properties derived from its rotaxane structure, which is frequently employed in molecular machines (Fig. 3).^21^ This gel differs from physical and chemical gels in that its crosslinks are designed to move freely. This gel is known as a slide-ring or topological gel. In this gel, polymer chains with bulky end groups are topologically connected by figure-eight crosslinks rather than covalent crosslinks (like those in chemical gels) or attractive interactions (like those in physical gels). These crosslinks can therefore move freely along the polymer chain, equalizing the tension in the polymer chain as though it were a pulley. Consequently, the gel exhibits tough mechanical properties. The collective action of the butyric acid molecular machines within the gel is reflected in its macroscopic properties.

**Fig. 4:**
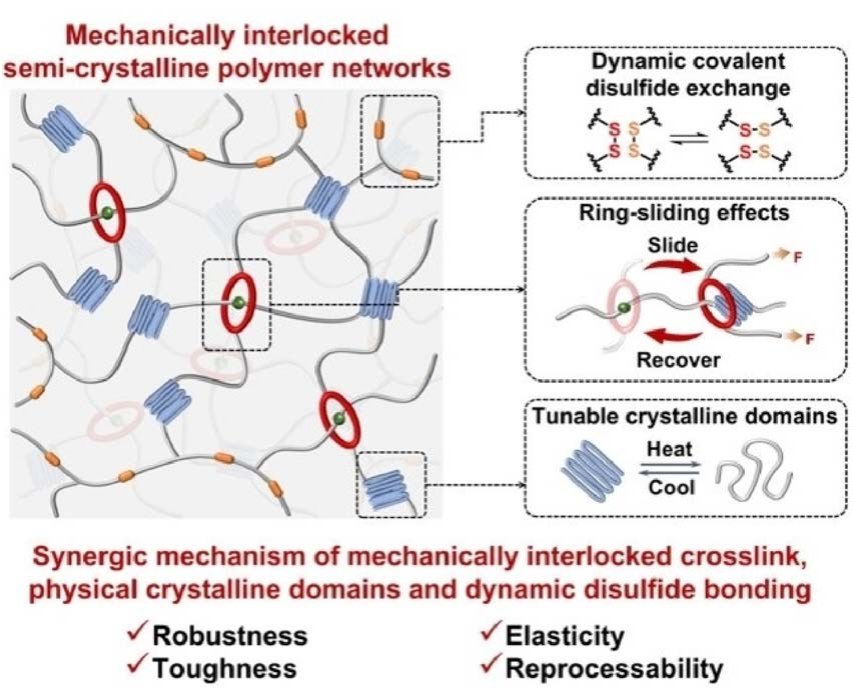
Mechanically interlocked semicrystalline networks containing rotaxanes, using 24-crown-8 ether as the interlocking crosslinking agent. Incorporating mechanical chemistry into conventional semicrystalline networks simultaneously enhances conventionally contradictory material properties such as mechanical toughness, strength and elasticity. Reprinted with permission from ref. 22 Copyright 2023 Wiley-VCH.

The mechanical properties of materials that utilize the collective behaviour of rotaxane molecular machines are currently being studied in more detail. Qu and co-workers constructed mechanically interlocked semicrystalline networks containing rotaxanes, using 24-crown-8 ether as the interlocking crosslinking agent (Fig. 4).^22^ Incorporating mechanical chemistry into conventional semicrystalline networks at once enhances conventionally contradictory material properties such as mechanical toughness, strength and elasticity. Mechanical toughness and strength result from the dissociation of crystalline domains through sieve-like macrocycles. The nanocrystalline domains and ring-sliding effect accelerate segmental motion and efficiently reduce energy dissipation. Elastic contraction of flexible segments within the amorphous polymer matrix is promoted, resulting in instantaneous recovery.

**Fig. 5:**
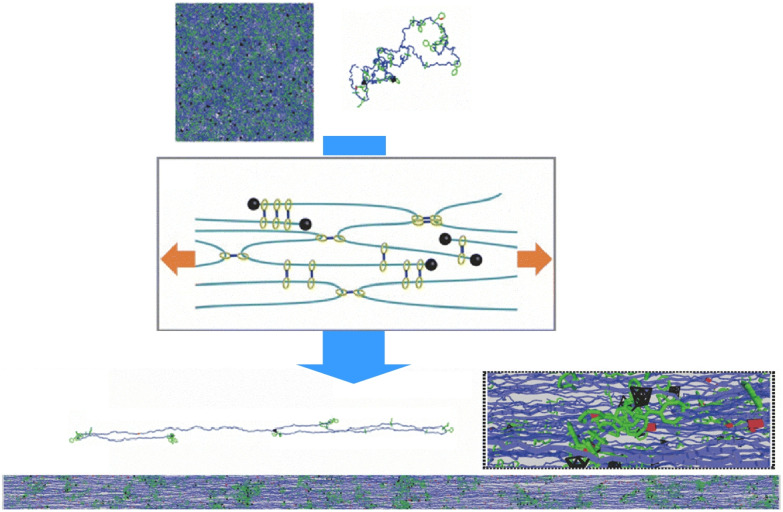
Slide-ring gels exhibiting uniform chain orientation and efficient stress distribution through the network. The distribution of network strand lengths changes with deformation and the chains reorganize into short and long sections. Reprinted with permission from ref. 23, Copyright 2025 Royal Society of Chemistry.

Yasuda, Mayumi, Ito and co-workers used coarse-grained molecular dynamics simulations to study the mechanical behaviour of slide-ring gels under large deformations.^23^ Under strain, these gels exhibit uniform chain orientation and efficient stress distribution through the network. When the gel is deformed, the distribution of network strand lengths changes. During stretching, the chains reorganize into short and long sections. Slide-ring crosslinks are anchored at the ends or kinks of the polymer chains, ensuring that only the long polymer strands remain oriented in the stretching direction (Fig. 5). This suppresses strain hardening at large strains and relieves stress concentrations, providing the molecular basis for unusually high toughness in slide-ring gels.

Mayumi, Ito and co-workers developed a polyethylene glycol hydrogel cross-linked with a moderate amount of polymer forming sliding rings.^24^ This gel uses a damage-free toughening strategy for hydrogels involving strain-induced crystallization. In slide-ring gels, where polyethylene glycol chains are highly oriented and in contact with each other under large deformations, crystallinity forms and melts with stretching and contraction. The result is both rapid recovery of nearly 100% of the extensional energy and excellent toughness, the latter being an order of magnitude greater than that of covalently cross-linked homogeneous polyethylene glycol gels.

This section has presented some examples of cooperative action of molecular machines within an assembly yielding increased motion and impact. With appropriate design and arrangement of molecules, the rotors can interlock with each other and act as gears. Furthermore, even without direct contact, rotaxane molecular machines located remotely can cooperate to control the properties of larger objects (such as gels) *via* polymer chains, achieving high performance not seen in conventional materials. Having considered assemblies in general terms in this section, the next section will examine the collective behaviour of molecular machines in structurally controlled materials and environments.

### Collective behaviour in MOF and COF nanospaces

Molecular machines require molecular space within which to move. One effective approach is to use a host material with ample nanospace, which allows multiple molecular machines to act in unison within the material. Porous materials are promising candidates for this reason. Metal–organic frameworks (MOFs)^25^ and covalent organic frameworks (COFs)^26^ are two materials that fulfil this requirement because they can be assembled while maintaining the structural precision of the nanospace. This section introduces research into the collective behaviour of molecular machines within MOFs and COFs.

**Fig. 6:**
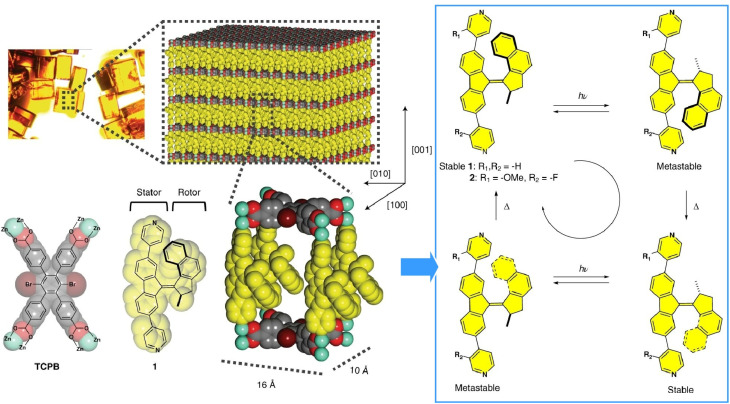
Molecular motors organized within MOFs, allowing them to operate at the same light-driven, unidirectional rotational speed (thermal helix reversal speed) in the solid state as they would in solution. Reprinted with permission from ref. 27, Copyright 2019 Springer-Nature.

Light-driven molecular motors can drive unidirectional rotation, and their behaviour in solution is well understood. However, Brownian motion complicates the precise nanoscale positioning necessary to harness cooperative action. Browne, Wezenberg, Feringa and co-workers demonstrated molecular motors organized within MOFs (Fig. 6).^27^ The motor units form part of an organic linker. Light-driven, unidirectional rotation of these motor units is preserved within the MOF structure. This enables the motors to operate in the solid state at the same rotational speed (thermal helix reversal speed) as they would in solution. The framework design ensures sufficient free volume, enabling the motor units within the pillars to perform 360° unidirectional rotation without hindrance. Potential applications include gas diffusion control, directional light-driven mass transport and miniature light-driven pumps in microfluidic devices.

In addition to enabling the free rotation of molecular rotors, the nanospace provided by MOFs can also be used to control the rotation. Several studies have been conducted to validate this idea. Feng, Xu, Tang and co-workers reported a system that incorporates an aggregation-induced luminescent rotor ligand into a MOF structure, known as (ZnETTB) (ETTB = 4′,4‴,4⁗′,4⁗″-(ethene-1,1,2,2-tetrayl)tetrakis(([1,1′-biphenyl]-3,5-dicarboxylic acid))).^28^ The β-benzene ring in the MOF framework has minimal rotational resistance and consequently rotates freely under excited-state conditions. Furthermore, incorporating *N*,*N*-diethylformamide as a guest ligand inhibits the rotation of the β-benzene ring. This limits the effect of intramolecular motion in the excited state, significantly enhancing luminescence.

**Fig. 7:**
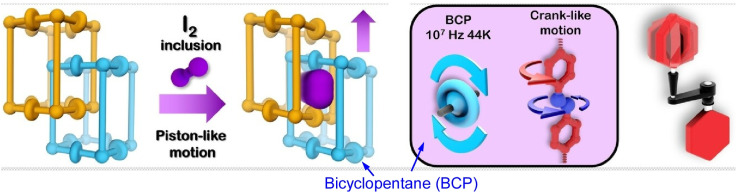
Bicyclopentane, two types of pyridyl rotor and an azo group responsible for pedalling motion organized in a flexible, double-interpenetrating pillar MOF. Adsorption of iodine vapor induces structural rearrangements, displacing two distinct subnetworks through cooperative piston-like motion. Reprinted with permission from ref. 29, Copyright 2024 Wiley-VCH.

Combining molecular rotors with optical functions and MOFs containing twisted aggregation-induced luminescent rotors is useful for sensing and detection applications. Multiple rotors engaging in collective behaviours within the MOF enhances the signal intensity sufficiently to meet thresholds. By engaging several mechanical components within the MOF, Bracco, Comotti and co-workers demonstrated that controlling the cooperative rotational motion of the MOF structure can enable the creation of molecular machines in the solid state.^29^ In a flexible, double-interpenetrating pillar MOF, they organized fast-moving components, including bicyclopentane, two types of pyridyl rotor and an E-azo group responsible for high-precision pedalling motion. Structural rearrangements induced by iodine vapor adsorption, for example, can displace two distinct subnetworks through cooperative piston-like motion (Fig. 7). Such designs could lead to the development of highly sophisticated interlocking mechanisms for molecular machines.

**Fig. 8:**
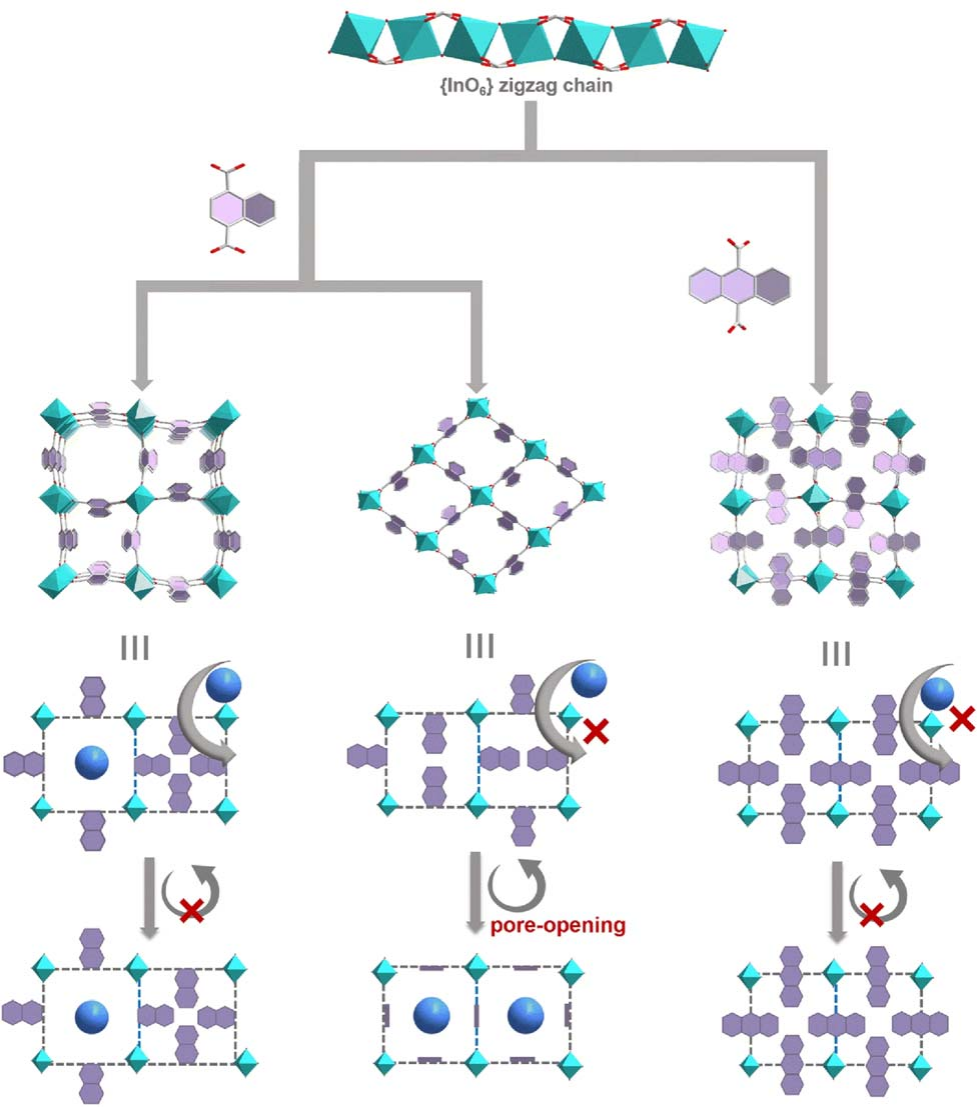
Molecular rotors with differing motional properties introduced into MOFs. Rotation of the molecular motors drives a gate-opening effect, precisely modulating the adsorption and separation of gas molecules. Reprinted with permission from ref. 31, Copyright 2024 Royal Society of Chemistry.

Various materials that incorporate molecular machine structures into MOF structures have been reported. For example, Chen, Zhu and co-workers constructed MOFs using mechanically linked [3]rotaxanes as ligands.^30^ The nanoconfinement effect of the macrocyclic structure keeps the flexible bis(*p*-phenylene) axis in a pseudo-rigid state. Coordination with Zn(ii) ions results in the formation of 2D and 3D MOFs. This approach of mechanically rigidifying flexible ligands sheds light on MOF design. Zhai and co-workers introduced molecular rotors into MOFs to investigate the controlled gate-opening effect of organic linker motors.^31^ Introducing molecular rotors with different motional properties into MOFs enhances their responsiveness, enabling precise control over the recognition, adsorption and separation of gas molecules (Fig. 8). This unique adsorption behaviour is driven by the gate-opening effect induced by rotation of the molecular rotors. For instance, electrostatic attraction between the framework and C_2_H_2_ molecules can greatly enhance C_2_H_2_ adsorption at low pressure, thereby improving C_2_H_2_ selectivity. Zhai and co-workers also demonstrated that controllable C_2_H_2_/CO_2_ adsorption separation can be achieved by introducing molecular rotors to adjust the pore environment of MOFs in a purposeful manner.^32^ Introducing smaller ligands increases the pore size, providing CO_2_ with more opportunities to interact with the 2D layers and strengthening its interaction with the framework, thereby enabling selective CO_2_ capture.

**Fig. 9:**
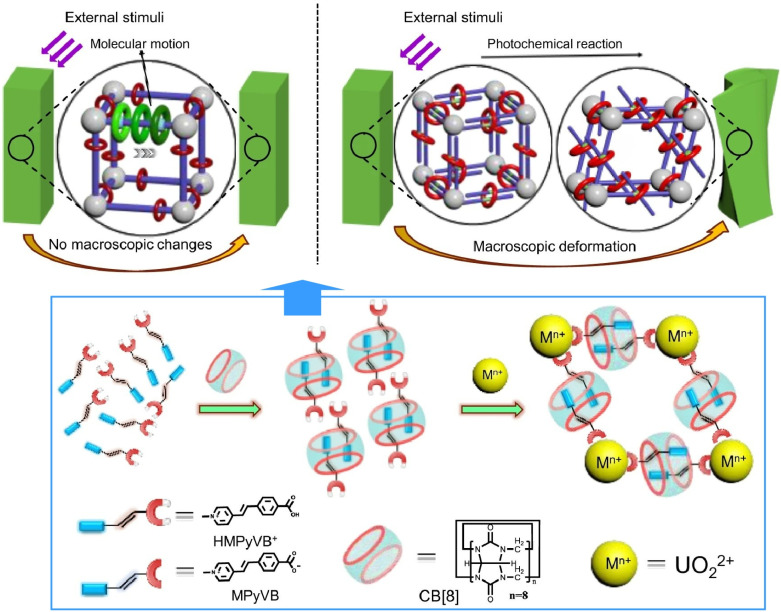
A photo-responsive metal–organic rotaxane framework composed of a cucurbit[8]uril-based macrocyclic pseudorotaxane linker and a photoactive styrene-derived guest molecule, (*E*)-4-[2-(methylpyridin-4-yl)vinyl]benzoic acid. The bending speed of this crystalline material is two orders of magnitude slower than that of other photoresponsive crystalline materials. Reproduced under terms of the CC-BY license from ref. 33, 2022 Springer-Nature.

MOF structures that incorporate molecular machines exhibit a variety of properties. Another interesting phenomenon is the ability to control the mechanical properties of MOF materials with the collective effects of molecular machines incorporated into MOFs. Mei, Feng, Shi and co-workers synthesized a photo-responsive metal–organic rotaxane framework using a cucurbit[8]uril-based macrocyclic pseudorotaxane linker and a photoactive styrene-derived guest molecule, (*E*)-4-[2-(methylpyridin-4-yl)vinyl]benzoic acid (Fig. 9).^33^ Due to the confinement effect of the macrocyclic structure and the role of the columnar structure in 3D lattice stacking, the bending speed of this crystalline material is two orders of magnitude slower than that of other photoresponsive crystalline materials. Consequently, the bending motion of the crystalline state can be precisely controlled. This paves the way for the development of molecular machine materials with microscopic and macroscopic dynamics, as well as stimuli-responsive behaviour. Li *et al.* reacted 4,4′,4′-triphenylamine tricarboxylate, which has a unique molecular rotor structure, with rare-earth metal ions to obtain dynamic luminescent MOF materials.^34^ Efficient transfer of absorption energy to the rare-earth metal ions *via* the antenna effect resulted in emission in the blue, yellow-green, red and near-infrared regions. These luminescent properties are useful for sensing substances. The detection limit for Al^3+^ ions was far below the maximum concentration specified by the US Environmental Protection Agency for drinking water. Similarly, the detection limit for H_2_O_2_ was much lower than the H_2_O_2_ content in cancer cells. Furthermore, Gd(iii)-containing MOFs exhibit singlet oxygen generation ability and blue ligand emission. Introducing aggregation-induced emission luminogens with molecular rotor structures improves the sensing performance of the luminescent MOFs, paving the way for dynamic, highly sensitive sensor materials.

**Fig. 10:**
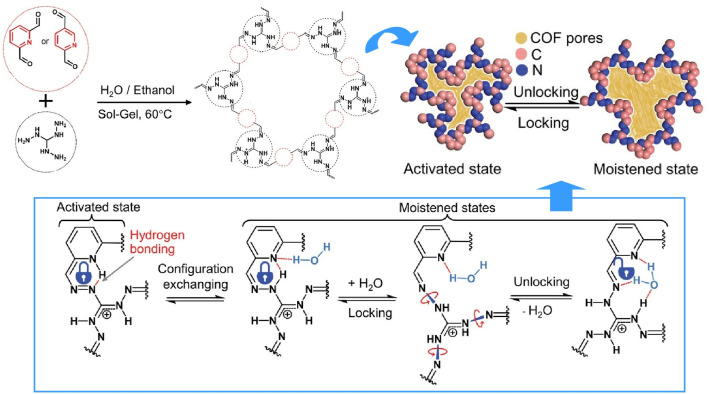
COF films with guest-dependent structural flexibility and moisture-driven actuation. This is enabled by intramolecular hydrogen bonds within the azine-pyridine molecular backbone that act as specific locks, opening and closing the N–N bonded molecular rotors in response to guest molecules. Reprinted with permission from ref. 35, Copyright 2024 Wiley-VCH.

Dynamic COFs are a promising platform for soft actuators. By incorporating dynamically operating molecular rotors into COFs, Pi and co-workers synthesized COF films with excellent guest-dependent structural flexibility and moisture-driven actuation (Fig. 10).^35^ These COF films possess intramolecular hydrogen bonds within the azine-pyridine molecular backbone that act as specific locks, opening and closing the N–N bonded molecular rotors in response to guest molecules. This guest-dependent structural transformation is modulated by the hydrogen bond between the pyridinyl N and guanidinium N, and integrating this COF film into a portable respiratory monitoring system could lead to diagnostic systems for respiratory diseases, such as sleep apnoea and asthma.

Porous materials such as MOFs and COFs exhibit regular porosity, providing nanospaces in which molecular machines can move freely. As the above examples demonstrate, large numbers of molecular machines can undergo collective actions within these materials. This property enables precise, molecular-level properties and functions to be translated into macro-level outputs. This is a highly rational strategy for expressing molecular-level phenomena as macroscopic functions while eliminating detrimental interference between components. The collective behaviour of molecular machines in MOFs and COFs demonstrates potential as a strategy for converting molecular functions into practical outputs.

### Action within crystals

The concept of confining molecular machines within the nanospace of MOFs while allowing them to move freely can be analogously applied in crystals. Designing crystal structures allows for the formation of porous MOFs and gives rise to a variety of regular structures. Considering the collective behaviour of molecular machines in regular structures, applying this concept to crystal structures is believed to be extremely meaningful. In this section, we will provide an overview of research examples demonstrating the collective behaviours of molecular machines in crystal structures.

**Fig. 11:**
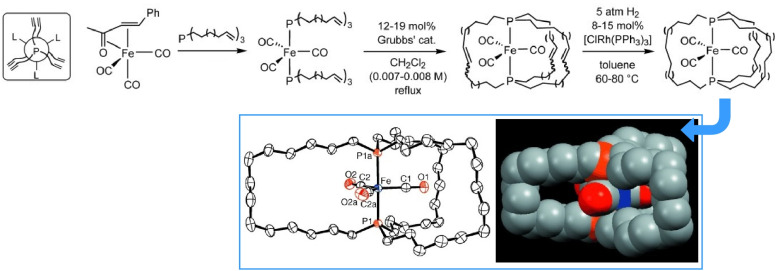
A crystal structure with symmetry, connectivity and rotor function akin to a molecular gyroscope. The stator comprises three spokes that span both ends of the gyroscope axis; adjusting the length of the spokes tunes the rotational barrier of the internal rotor. Reprinted with permission from ref. 36 Copyright 2004 Wiley-VCH.

Gladysz *et al.* reported a crystal structure that effects the symmetry, connectivity and rotor function of molecular gyroscopes (Fig. 11).^36^ The rotor can be either an {Fe(CO)_3_} group or an isoelectronic and isosteric {Fe(CO)_2_(NO)}^+^ group. The stator comprises three spokes that span both ends of the gyroscope axis. Adjusting the length of the spokes tunes the rotational barrier of the internal rotor. Molecular systems with controlled mobility have also been integrated into liquid crystal structures.

**Fig. 12:**
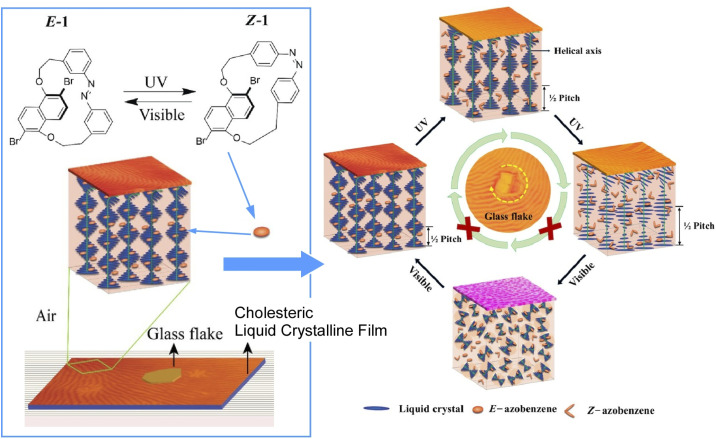
A chiral nematic liquid crystal containing a chiral azobenzene derivative molecular system that exhibits reversible EZ photoisomerization accompanied by significant changes in helical twisting power. Reprinted with permission from ref. 37, Copyright 2019 American Chemical Society.

Tamaoki and co-workers developed a chiral nematic liquid crystal containing a chiral azobenzene derivative molecular system that exhibits reversible EZ photoisomerization accompanied by significant changes in helical twisting power (Fig. 12).^37^ Alternating cycles of UV and visible light irradiation result in multiple rotations of a piece of glass resting on this liquid crystal layer in the same direction. The rotation of the piece of glass on the liquid crystal film is controlled by the photo-isomerisation and orientation of the embedded chiral, photo-responsive dopant molecules. This can be viewed as the continuous conversion of light energy into mechanical work. It could contribute to the development of molecular machines that can convert light energy into work.

**Fig. 13:**
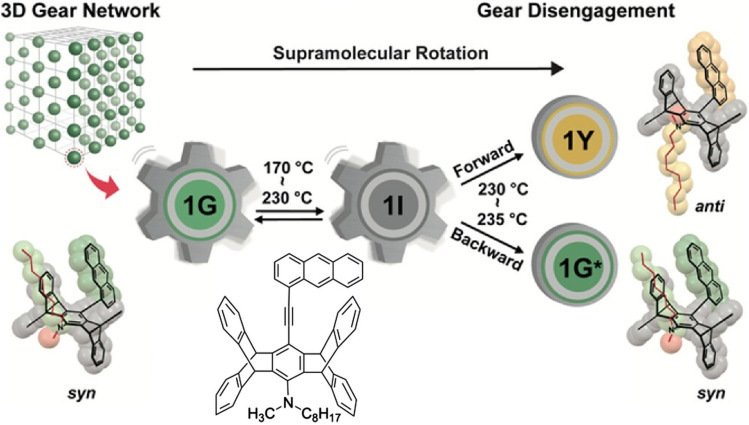
A 3D supramolecular gear network in the green-emitting polymorph of a dialkylamino-substituted anthracene-pentiptycene π-system undergoing unusual, bifurcated polymorphic transitions to a yellow-emitting polymorph and a different green-emitting polymorph *via* 3D correlated supramolecular rotation. Reproduced under the terms of the CC-BY license from ref. 38, 2024 American Chemical Society.

Yang *et al.* demonstrated the feasibility of constructing a 3D molecular gear system (Fig. 13).^38^ They observed a 3D supramolecular gear network in the green-emitting polymorph of a dialkylamino-substituted anthracene-pentiptycene π-system undergoing unusual, bifurcated polymorphic transitions to a yellow-emitting polymorph and a different green-emitting polymorph *via* 3D correlated supramolecular rotation. Their work has shown a correlation between crystal structure and solid-state molecular dynamics. This demonstrates how the 3D molecular gear system can efficiently transfer thermal energy in order to drive polymorphic transitions, as well as inducing fluorescent chromism through remarkable conformational and packing changes.

Jin and co-workers are developing various molecular machines that operate within a crystalline matrix. In their review article, ‘Rotational dynamics in crystalline molecular machines’, they examine the relationship between solid-state molecular rotational motion and photophysical properties, such as fluorescence, phosphorescence, and chiroptical response.^39^ Advances in crystalline molecular rotors, also known as solid-state molecular machines, allow for the precise modulation of molecular dynamics and demonstrate novel approaches to controlling photophysical behaviour. In a perspective article titled ‘Design of molecular crystals towards crystalline molecular machines: rotors, gears, and motors’, they focus on designing crystalline molecular materials that facilitate controlled molecular motion.^40^ They specifically mention three major types: rotors, gears, and motors. Most existing architectures are based on the dichotomy between dynamic and static components—namely, rotors and stators. However, the potential for more complex and emergent behaviours to arise from collective dynamics and distributed activity must be explored.

Bridging the gap between molecular-level design and long-range crystalline order could open up new avenues for developing molecular-based dynamic crystalline materials with engineered mechanical responses. Crystalline molecular gear systems, in particular, give way to a new design paradigm by enabling mechanically correlated motion between multiple molecular components. These systems exhibit synchronized or coordinated rotational motion through interdigitated structures, which can be directed and regulated by crystal packing and intermolecular forces. Examples of unidirectional rotation and photoinduced gearing suggest that directional control is possible even in rigid crystalline environments. Integrating responsive optical, electronic or mechanical properties and motion will be important in developing next-generation smart materials. Crystalline molecular machines will enable future applications in adaptive photonic devices, mechanical actuators and energy conversion systems.

**Fig. 14:**
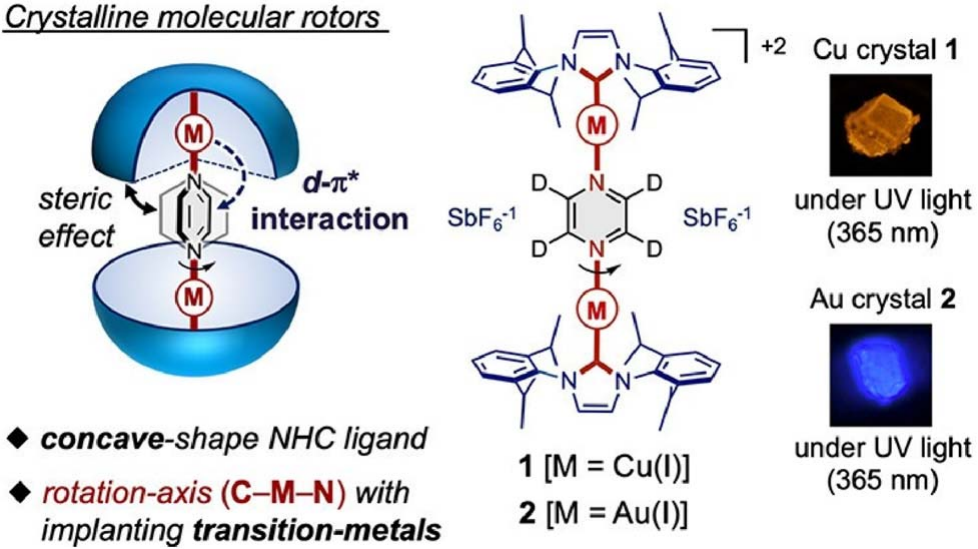
A luminescent crystalline molecular rotor, in which a central pyrazine rotor is connected by an implanted transition metal (Cu or Au) to an easily accessible enclosure formed by two N-heterocyclic carbenes in a discrete binuclear complex. Reprinted with permission from ref. 41, Copyright 2021 American Chemical Society.

Some examples of molecular rotors in different crystal structures are presented below. Jin, Ito and co-workers reported a novel design for a luminescent crystalline molecular rotor (Fig. 14), in which a central pyrazine rotor is connected by an implanted transition metal (Cu or Au) to an easily accessible enclosure formed by two N-heterocyclic carbenes in a discrete binuclear complex.^41^ They designed a semi-closed, encapsulated, bimetallic complex rotor with a concave, bulky *N*-heterocyclic carbene stator and an aromatic rotor that is coaxially coordinated to the transition metal along the rotation axis. This design enables the molecular rotor to rotate within the crystal. Furthermore, the activation energy of rotation can be adjusted by altering the implanted metal. The packing structure effect, which is often important in many amphiphilic molecular crystals, is absent. Based on the examples presented here, a general method can be developed that uses abundant ligands as functional stators and metal–ligand coordination bonds as tuneable rotation axes in amphiphilic crystals. This would greatly facilitate the development of new functional materials based on changes in molecular dynamics in static crystalline solids. Additionally, Ito, Jin and co-workers demonstrated the solid-state rotational motion of two larger molecules (triptycene and pentiptycene) by encapsulating bulky N-heterocyclic carbene Au(i) complexes in crystalline media.^42^ This enables further study and design of systems in which large, complex molecular units can perform rotational motion while maintaining the structural integrity of the system and the mobility of its components.

**Fig. 15:**
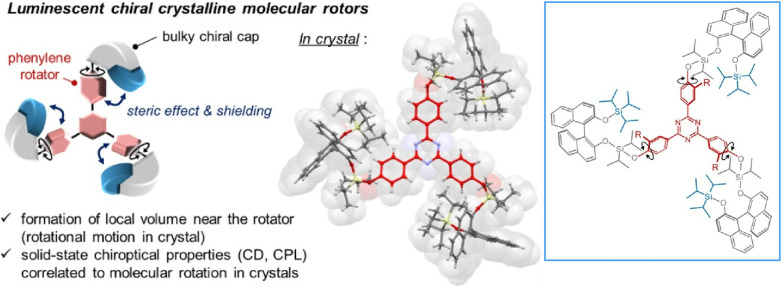
Chiroptical properties correlated with rotational motion in crystalline media achieved by incorporating a bulky chiral cap into triaryltriazine. Reprinted with permission from ref. 43, Copyright 2024 American Chemical Society.

Jin and his team developed a new structural motif for luminescent, chiral, crystalline molecular rotors that exhibit chiroptical properties correlated with rotational motion in crystalline media (Fig. 15).^43^ By incorporating a bulky chiral cap into triaryltriazine, the chiroptical properties became correlated with rotational motion in the crystalline state. These steric rotors exhibit remarkable circularly polarized fluorescence, which is induced by the suppression of molecular motion in an excited state with a sterically constrained lattice environment. The bulky chiral cap was obtained by introducing a triisopropylsilyl group into the axially chiral binaphthyl group. Molecular rotation promotes the structural relaxation of the bulky, chiral-capped triaryltriazine in the excited state upon light absorption. Furthermore, phenylene rotation in the crystal enhances efficient intersystem crossing through the formation of a stable triplet state. These results demonstrate a useful molecular design for modulating solid-state chiroptical properties through rotational motion. It may be possible to tune the energy gap between the singlet and triplet states, which is generally considered difficult to achieve in crystalline media.

**Fig. 16:**
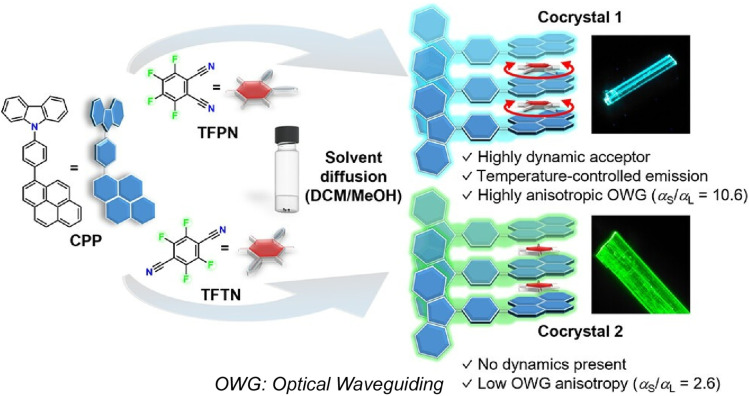
Optical waveguiding crystals featuring large anisotropy and fluorinated acceptor molecules with in-plane rotational dynamics where the charge transfer co-crystal exhibits anisotropy due to the orientation of the transition dipole moment, as well as high reabsorption due to crystal packing. Reproduced under the terms of the CC-BY license from ref. 44, 2025 American Chemical Society.

Jin, Hayashi, Rodríguez-Molina and co-workers created binary optical waveguiding crystals featuring large anisotropy and fluorinated acceptor molecules with in-plane rotational dynamics (Fig. 16).^44^ The charge transfer co-crystal exhibits anisotropy due to the orientation of the transition dipole moment, as well as high reabsorption due to crystal packing. Notably, the substantial linear increase in photoluminescence at low temperatures can be attributed to a reduction in in-plane rotational motion at these temperatures. These findings suggest that the co-crystal possesses excellent binary optical waveguiding properties and thermally driven photoluminescence, making it suitable for use in optically controlled gates, for example.

**Fig. 17:**
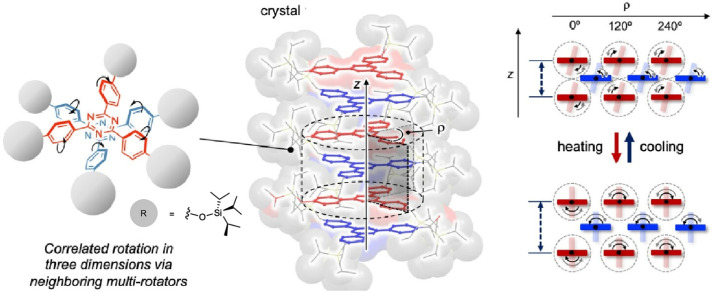
Stacked clutch structure formed in the crystalline phase by a triaryltriazine rotor bearing bulky silane groups in the *para*-position of the peripheral phenylene groups. The crystalline rotor's phenylene units can perform two distinct, interconvertible, correlated molecular motions. Reprinted with permission from ref. 45, Copyright 2023 American Chemical Society.

To demonstrate gear-shift functionality at the molecular level, Jin, Ito and co-workers investigated a columnar, stacked clutch structure formed in the crystalline phase by a triaryltriazine rotor bearing bulky silane groups in the *para*-position of the peripheral phenylene groups (Fig. 17).^45^ The crystalline rotor's phenylene units can perform two distinct, interconvertible, correlated molecular motions. These intermolecular rotational motions can be switched *via* a thermally induced phase transition between the crystals. The intermolecular stacking of C3-symmetric rotors, driven by the steric repulsion of the bulky stator, is a promising strategy for generating various correlated molecular motions in the crystalline phase. Reversible modulation of these correlated rotations is achieved *via* a thermally induced inter-crystalline phase transition. This can function as a gear shift at the molecular level.

Unlike MOFs, crystal structures do not provide nanospace; however, they do provide an ordered structure in which molecular machines can perform collective behaviours. These structures allow molecular machines to move freely and can create systems in which multiple machines work together. It is also noteworthy that materials can be constructed in which molecular gear structures, comprising interlocking molecular rotors, can be integrated. This allows movement at the molecular level to influence the properties of macroscopic materials, and further advancements in this strategy are anticipated.

### Collective behaviour on solid surfaces

Although many techniques exist for analysing the behaviour of molecular machines, the most direct method is to visualize them through molecular imaging. These analyses are typically performed using either scanning tunnelling microscopy (STM) or atomic force microscopy (AFM).^46^ This is achieved by placing the molecular machine on a solid substrate. In this section, we present examples of research into the behaviour of molecular motors and their associated gears at solid interfaces.

**Fig. 18:**
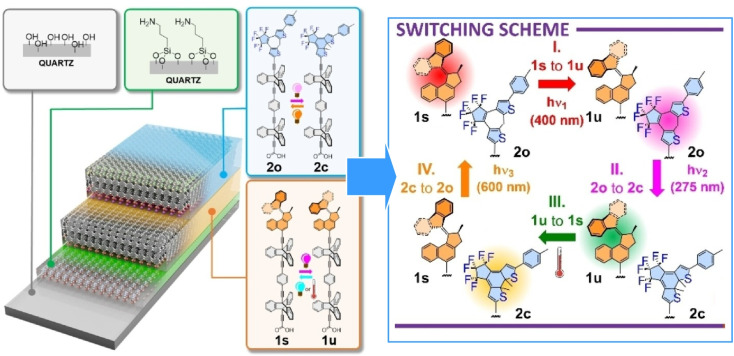
A monolayer system of optically switched rod-like molecules incorporating a diarylethene-based switch and a unidirectional, light-driven molecular motor on a solid substrate built on a triptycene-based molecular pedestal. Reprinted with permission from ref. 47, Copyright 2024 Wiley-VCH.

As demonstrated through the examples of MOFs and crystals mentioned above, deliberate molecular design can enable molecular machines to function independently within thin films on solid substrates. Kaleta *et al.* fabricated a monolayer system of optically switched rod-like molecules incorporating a diarylethene-based switch and a unidirectional, light-driven molecular motor on a solid substrate (Fig. 18).^47^ This system is built on a triptycene-based molecular pedestal. The film was then smoothly transferred onto gold and quartz surfaces using the Langmuir–Blodgett (LB) technique. Repeated deposition yielded bilayer systems containing one layer with a diarylethene-based optical switch and another with a unidirectional, light-driven molecular motor. It was confirmed that the optically switched molecular machine maintained full functionality while operating at the solid–gas interface. By using appropriate monochromatic light, layers composed of the same type of optical switch can be selectively targeted. This could potentially be utilized to construct new types of memory devices boasting molecular level multilayer structures.

**Fig. 19:**
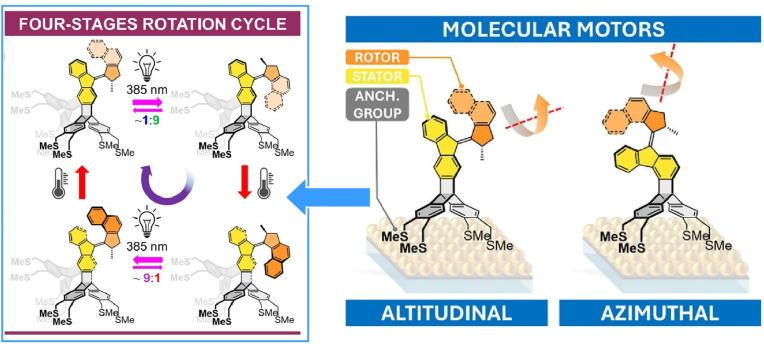
Two light-driven molecular motors fused to a triptycene-based tetrapodal platform, with their rotational axes oriented either parallel or perpendicular to the surface. Even when assembled as monolayers on gold surfaces, the motors maintained full rotational functionality, demonstrating the tetrapodal platform's ability to minimize surface interactions. Reproduced under the terms of the CC-BY license from ref. 48, 2026 Wiley-VCH.

Additionally, Kaleta *et al.* synthesized two light-driven molecular motors fused to a triptycene-based tetrapodal platform, with their rotational axes oriented either parallel or perpendicular to the surface (Fig. 19).^48^ Even when assembled as monolayers on gold surfaces, the motors maintained full rotational functionality, demonstrating the tetrapodal platform's ability to minimize surface interactions. This shows that the tetrapodal platform effectively decouples the chromophore from the metal surface, making it a promising candidate for developing advanced surface-mounted molecular machines.

**Fig. 20:**
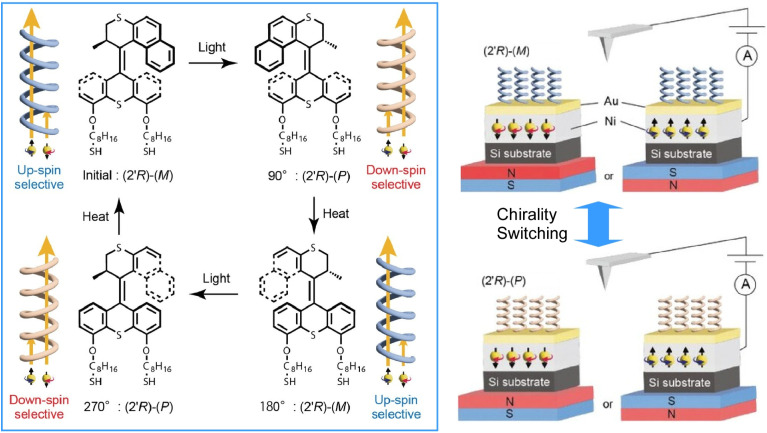
Self-assembled monolayers of molecular motors based on overcrowded alkenes. The chirality of the molecules can be switched by forming covalent bonds between the molecules and electrodes, allowing the direction of spin polarization to be manipulated externally with remarkable stability and repeatable control. Reprinted with permission from ref. 49, Copyright 2023 Wiley-VCH.

Chiral molecules have recently attracted renewed attention as a highly efficient source of spin-selective charge release, resulting from a phenomenon known as ‘chiral-induced spin selectivity’. Noting this, Sato, Yamamoto and co-workers developed self-assembled monolayers of molecular motors based on overcrowded alkenes (Fig. 20).^49^ By forming covalent bonds between electrodes and the molecules, their chirality can be switched, allowing the direction of spin polarization to be manipulated externally with remarkable stability and repeatable control. Thiol-terminated alkyl chains (C_8_) provide the necessary space for the structure to rotate unidirectionally.

**Fig. 21:**
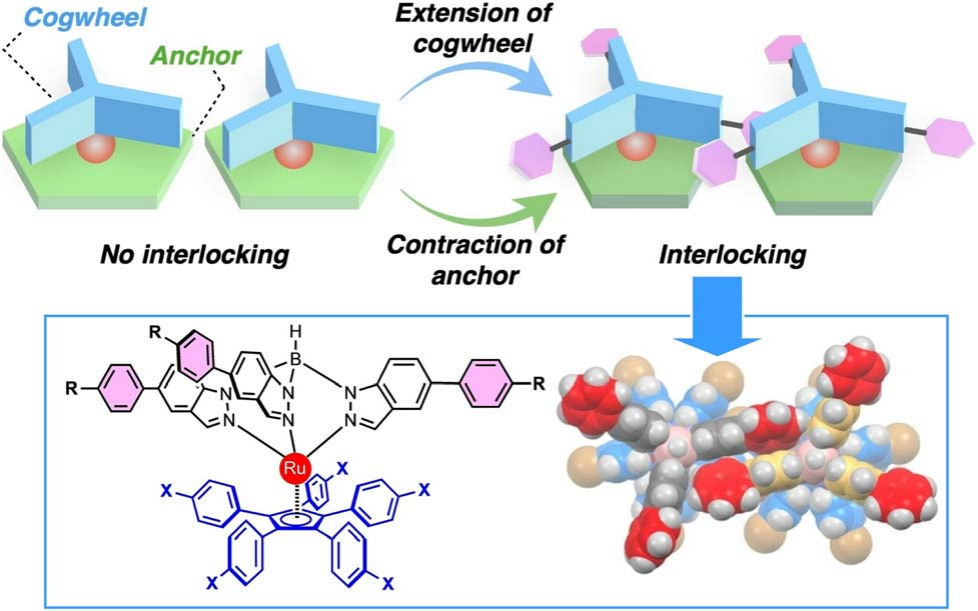
A ruthenium-based molecular gear consisting of a tripodal ligand with a pentaphenylcyclopentadienyl ligand as the anchor and aryl-extended indazole as the teeth. Reproduced under the terms of the CC-BY license from ref. 50, 2023 Wiley-VCH.

Researchers have also attempted to design molecular gears that intermesh with molecular rotors *via* attractive van der Waals interfaces, which are essential for forming highly ordered assemblies. Rapenne and co-workers designed and synthesized a ruthenium-based molecular gear consisting of a tripodal ligand with a pentaphenylcyclopentadienyl ligand anchor and aryl-extended indazole teeth (Fig. 21).^50^ The single-crystal structure of the ruthenium complex reveals that the appended aryl groups increase the apparent diameter of the gear, making it larger than the anchor unit. Consequently, when the complex adsorbs to a surface, intermolecular gearing motion becomes favourable. The linear arrangement of the complex's tripodal ligands appears to interdigitate, providing efficient propagation of rotational motion.

**Fig. 22:**
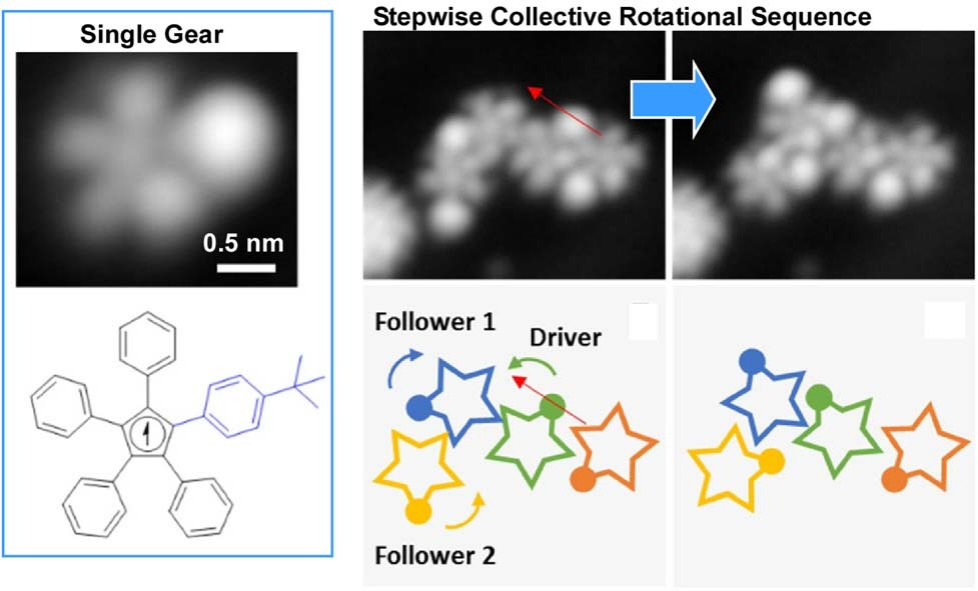
Manipulating the *tert*-butyl tooth of one gear with the STM tip induces steps in the rotational transmission between three interlocking gears. Small lateral displacements were observed in all three gears per rotational step. Reprinted with permission from ref. 51, Copyright 2020 American Chemical Society.

Some studies have observed the motion of interlocking molecular gears and molecular rotors. To realize a molecular gear train operating at the tip of an STM, each molecule must be stably anchored to a metal surface. In the case of the molecular gears demonstrated by Moresco *et al.*, placing a *tert*-butyl group on the end of one gear tooth was beneficial for both tip-induced manipulation and rotation monitoring.^51^ Using this optimized molecule, they achieved reproducible, stepwise rotation of individual gears and transmitted rotation to up to three interlocking units (Fig. 22). Manipulating the *tert*-butyl tooth of one gear with the STM tip enabled them to induce steps in the rotational transmission between three interlocking gears. Counterclockwise rotation of the driver caused the first follower to rotate clockwise by 75°, while simultaneously causing another follower to rotate counterclockwise by 78°, for a total of 104°. In the aftermath of a rotation step, small lateral displacements were observed in all three gears. The ability to control the rotation of multiple gears within a gear train is crucial for the development of molecular machines, such as mechanical computers.

**Fig. 23:**
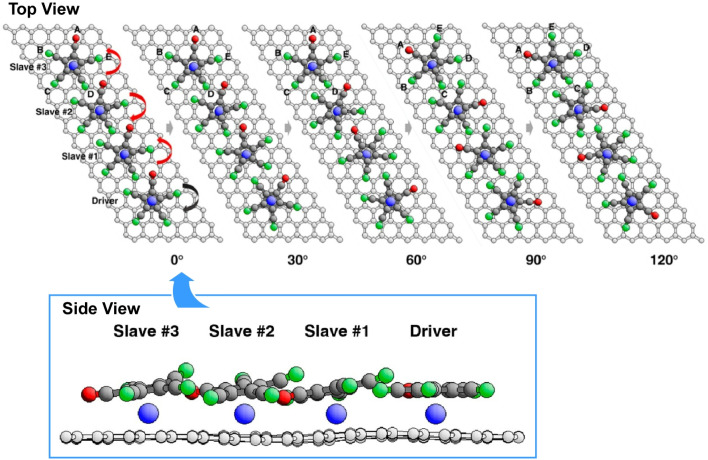
Transmission of rotational motion and energy over longer distances through chains of passive, gear-like ‘slave’ molecules. Ensuring that the slave molecules are fully relaxed at each imposed driver rotation angle enables them to follow energy-optimized paths involving rotation, tilting, bending, sliding and disassembly. Reprinted with permission from ref. 52, Copyright 2018 American Chemical Society.

Van Hove and co-workers explored the transmission of rotational motion and energy over longer distances through chains of passive, gear-like ‘slave’ molecules (Fig. 23).^52^ Ensuring that the slave molecules are fully relaxed at each imposed driver rotation angle enables them to follow energy-optimized paths involving rotation, tilting, bending, sliding and disassembly. The quasi-classical picture of these rotations is realistic and intuitive at a qualitative level. Therefore, neither prediction of the general behaviour of gear rotations nor gear couplings require quantum mechanics. Nevertheless, quantum effects, such as the height of rotational and translational barriers, underlie these motions at a quantitative level. Quantum tunnelling, thermal effects and energy dissipation also play a significant role. This research will aid the design of gear systems and contribute to the development of nanoscale molecular robots.

**Fig. 24:**
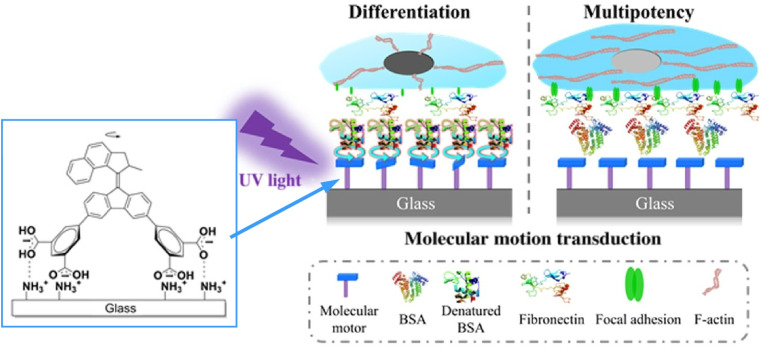
Artificial rotary molecular motors on a solid surface can exert motion to control adsorbed protein layers, ultimately determining the fate of human bone marrow-derived mesenchymal stem cells (hBM-MSCs). Reprinted with permission from ref. 53, Copyright 2020 American Association for the Advancement of Science.

The collective action of molecular machines on solid surfaces can be employed to control larger, more sophisticated material systems. For instance, cell differentiation can be regulated through the collective activity of molecular machines. Feringa, van Rijn and co-workers demonstrated that the collective dynamic motion of a layer of artificial rotary molecular motors to UV light can inactivate adsorbed protein layers at a biological level. Since stem cells on dynamically altered surfaces are more likely to differentiate into osteoblasts than those on static surfaces, this ultimately determines the fate of human bone marrow-derived mesenchymal stem cells (hBM-MSCs) (Fig. 24).^53^ Surfaces on which molecular motors engage in collective behaviours offer various opportunities for mechanical stimulation, controlling cell fate, and creating responsive biomimetic materials. This paves the way for synthetic molecular motors to be used in biomedical and clinical applications.

In summary, the shape and movement of molecular machines can be directly observed on solid surfaces. Molecular gears in which molecular rotors intertwine and move in unison as part of a sophisticated collective behaviour have been demonstrated. By observing molecular structures using STM and performing theoretical calculations, it is also possible to study the operating behaviour of molecular machines at the quantum level. Of particular note is how cell fate can be controlled by the collective rotation of molecular motors on surfaces. This aligns with the previously discovered paradigm whereby cells receive stimuli from surfaces and undergo various changes. Surfaces and other environments bridge molecular machine movement with macroscopic phenomena.

### Collective behaviour at liquid interfaces

The final example in the previous section demonstrated that interfacial environments are ideal for integrating the operation of molecular machines with macroscopic functions. This property is particularly pronounced at liquid interfaces, where the interface itself is dynamic. For instance, a monolayer at the air–water interface has macroscopic dimensions in the in-plane direction, juxtaposed by its nanomolecular thickness. In such an environment, macroscopic perturbations applied in the in-plane direction can be linked to molecular functions within the thickness of the film. Thus, macroscopic behaviour and nanoscale functions can be linked at the liquid interface.^54^ Integrating molecular machines as a monolayer at the air–water interface makes it possible to control their collective behaviour through macroscopic manipulation.^55^

**Fig. 25:**
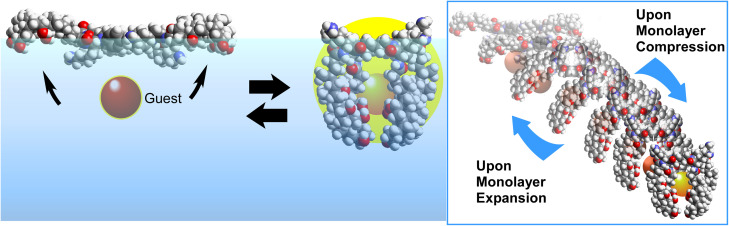
A steroid cyclophane molecular machine with a central ring structure of cyclophane and four rigid plate-like steroid moieties to grasp and capture guest molecules upon compression of its monolayer at the liquid interface.

The first molecular machine to be driven through macroscopic mechanical manipulation was a steroid cyclophane (Fig. 25).^56^ This machine consists of a central ring structure, or cyclophane, to which four rigid, plate-like steroid moieties are connected *via* flexible spacers. When compressed laterally to reduce its molecular area, this monolayer forms a cavity-like structure. Using this property, the molecular machine can grasp and capture guest molecules. Mechanical displacements of several tens of centimetres induce structural changes in the molecular machine at the nanometre level, enabling flexible control over the capture and release of molecules. This process can be used to fine-tune the structure of molecular receptors.

**Fig. 26:**
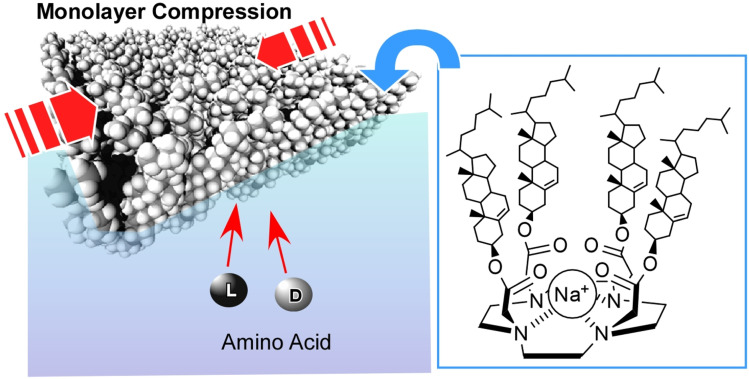
A cholesterol-armed cyclen with an asymmetric centre that can alter its environment by twisting was used as a molecular receptor for recognition of chiral amino acids with various surface pressures at the air–water interface.

A cholesterol-armed cyclen with an asymmetric centre that can alter its environment by twisting was used as a molecular receptor at the air–water interface (Fig. 26).^57^ The monolayer was then compressed gradually, and recognition of chiral amino acids at various surface pressures was tested. For example, when valine is used as a guest molecule, the d-isomer is recognized preferentially at low surface pressure; however, this preference reverses at high surface pressure, favouring the l-isomer instead. In other words, adjusting the conformation of the receptor molecule at the interface can reverse the chiral selectivity, even when using the same receptor molecule.

**Fig. 27:**
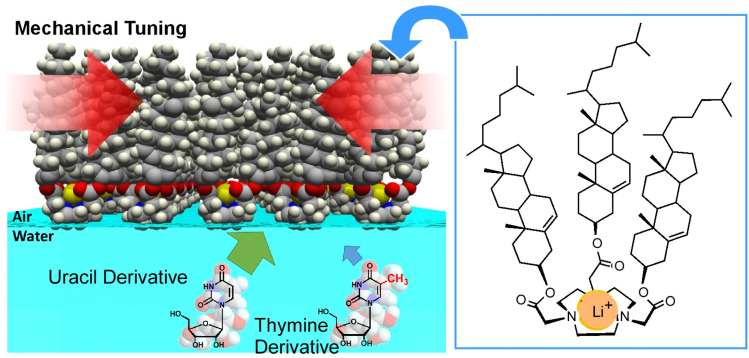
Cyclononane molecules containing multiple carbonyl and tertiary amino groups arranged on the water surface with the capability to discriminate between uracil and thymine derivatives at the air–water interface.

Similar tuning of molecular recognition properties has also been demonstrated in the recognition of nucleobases. In this case, cyclononane molecules containing multiple carbonyl and tertiary amino groups are arranged on the water surface and gradually deformed by pressure in order to adjust the hydrogen-bonding structure (Fig. 27).^58^ Despite the structural difference between uracil and thymine derivatives being merely a single methyl group, recognition was achieved at a rate 70- to 80-fold higher under the same conditions. This is impressive as even nucleic acids such as DNA and RNA fail to distinguish between thymine and uracil. Mechanical tuning is a very useful tool to find optimal structures for molecular recognition at interfaces.

**Fig. 28:**
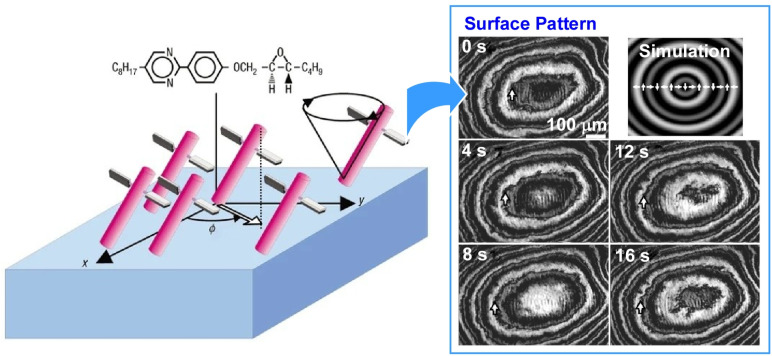
A monolayer of simple, rod-shaped molecules with chiral propellers (left) exhibiting a spatiotemporal pattern of molecular orientation that closely resembles the target pattern observed in the Belousov–Zhabotinsky reaction (right). Reprinted with permission from ref. 59, Copyright 2003 Springer-Nature.

Molecular motors are susceptible to thermal fluctuations at liquid interfaces due to their small size, which limits their ability to exert useful functions. Instead, a more feasible strategy is to condense molecular motors into soft, ordered phases (such as liquid crystals), achieving functionality through collective molecular driving. Tabe and Yokoyama demonstrated that a chiral liquid crystal monolayer spread on a glycerol surface functions as a condensed layer of molecular rotors undergoing coherent precession, driven by the movement of water molecules across the membrane (Fig. 28).^59^ This monolayer consists of simple, rod-shaped molecules with chiral propellers and exhibits a spatiotemporal pattern of molecular orientation that closely resembles the ‘target pattern’ observed in the Belousov–Zhabotinsky reaction. Reversing either the molecular chirality or the direction of water molecule movement can reverse the rotational direction of the transition from an expanding to a converging target pattern. Although the rotational motion of a single molecular motor is likely to be overwhelmed by thermal noise, the collective, cooperative motion of molecular motors, enabled by liquid crystal ordering, has been shown to amplify tiny individual motions into macroscale motion.

**Fig. 29:**
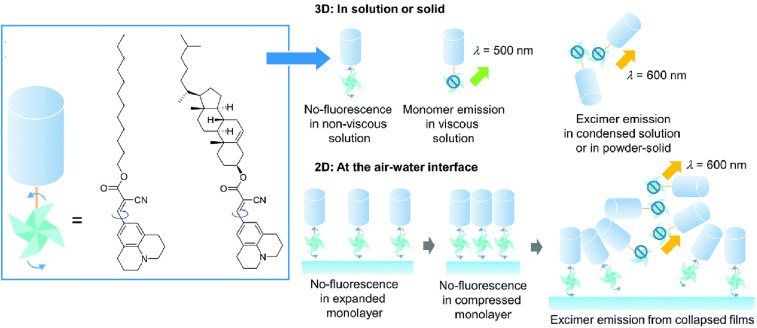
Behaviours of a 9-(2-carboxy-2-cyanovinyl)julolidine derivative, as a twisted intramolecular charge transfer molecular rotor. The intramolecular rotation of this molecular rotor is different as part of 2D and 3D collapsed monolayers at the air–water interface. Reprinted with permission from ref. 60, Copyright 2018 Royal Society of Chemistry.

As observed in crystals, the degree of rotational freedom of molecular rotors is dependent on the available free space. In monolayers on water surfaces and their collapsed three-dimensional structures, the rotational behaviour of molecular rotors depends on the packing of molecular aggregates. From this perspective, the behaviour of a 9-(2-carboxy-2-cyanovinyl)julolidine derivative, as a twisted intramolecular charge transfer molecular rotor, was investigated using *in situ* fluorescence spectroscopy (Fig. 29).^60^ This research examined the intramolecular rotation of molecules within 2D and 3D collapsed monolayers at the air–water interface. In solution, this molecular rotor exhibited suppressed molecular rotation and increased fluorescence intensity with increasing solvent viscosity. In contrast, monomer emission from the monolayer was weaker, suggesting that intramolecular rotation is not suppressed, even in densely ordered monolayers. Furthermore, fluorescence spectroscopy of Langmuir–Blodgett (LB) films revealed that molecular rotation remains unaffected even when monolayers are transferred to solid substrates. However, excimer emission due to aggregation was observed when the monolayer collapsed into a disordered 3D structure. If a 2D ordered structure can be maintained in a soft aggregate rather than a crystal, sufficient free space will be available to enable collective behaviour of the molecular rotors.

**Fig. 30:**
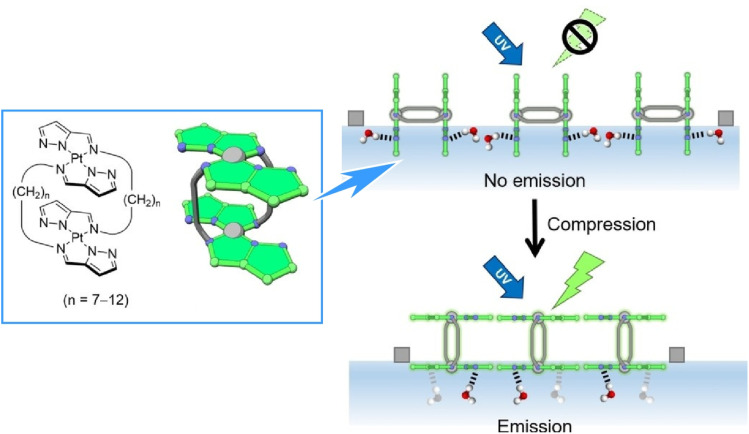
Orientation and luminescence properties of a double-paddle dinuclear Pt^II^ complex containing pyrazole rings linked by an alkyl spacer, which are dynamically manipulated at the air–water interface. Monolayer compression triggers a unique ‘submarine luminescence’ effect. Reprinted with permission from ref. 61, Copyright 2020 Wiley-VCH.

The air–water interface is a boundary between two media with a large difference in dielectric constant. Consequently, these media can exhibit significant differences in physical properties, especially engineering properties. Therefore, differences in molecular conformation and orientation can result in molecular machine-like functions. Fig. 30 shows an example of this, depicting the orientation and luminescence properties of a double-paddle dinuclear Pt^II^ complex containing pyrazole rings linked by an alkyl spacer being dynamically manipulated at the air–water interface.^61^ During mechanical compression, the complex emerges from water, simultaneously changing the orientation of the Pt^II^ complex planes from perpendicular to parallel and exhibiting a unique ‘submarine luminescence’ effect. The complex's phosphorescence is quenched at the air–water interface before monolayer formation; then, the intensity increases rapidly during monolayer compression. In submerged mode, the H-shaped dinuclear Pt^II^ complex sinks in water, resulting in weak luminescence expression due to hydrogen bonding. Mechanical compression then pushes the H-shaped complex to the surface, triggering transition to the floating mode where the Pt^II^ complex planes behave independently at the air–water interface. The surface in the air phase is unaffected by the dispersion of excitation energy due to molecular contact in the water phase. In other words, this molecular monolayer behaves as an assembly of molecular submarines.

**Fig. 31:**
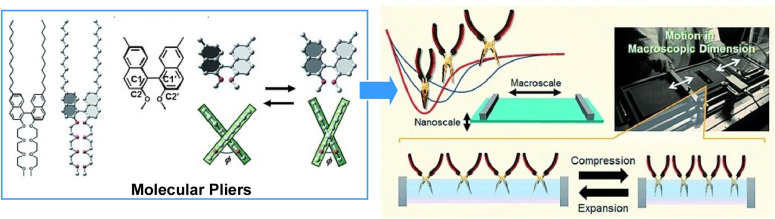
Binaphthyl-type amphiphilic molecules used as openable molecular pliers to examine patterns in macroscopic mechanical energy expenditure for molecular deformation. The pliers were arranged at the air–water interface and gradually compressed, and correlated with the measured dihedral angle of the binaphthyl group. Reprinted with permission from ref. 62, Copyright 2015 Wiley-VCH.

The operational characteristics of molecular machines that are collectively driven at the air–water interface have also been investigated. One topic of discussion has been the conversion of mechanical energy. To this end, molecular pliers were used as their movement is easy to assess and evaluate (Fig. 31).^62^ Binaphthyl-type amphiphilic molecules were used as openable molecular pliers to examine patterns in macroscopic mechanical energy expenditure to produce molecular deformation. First, the pliers were arranged at the air–water interface and gradually compressed, and the dihedral angle of the binaphthyl group was measured. Based on this data, the energy required for molecular deformation was calculated using quantum chemical modelling. Furthermore, the energy involved in the mechanical deformation of a macroscopic monolayer was estimated thermodynamically. The two values were very similar. As surface pressure increases from 0 to 10 mN m^−1^, approximately 0.2 kcal mol^−1^ of mechanical energy is stored and the same amount of molecular deformation energy is used. In contrast, at higher pressures, the molecular deformation energy becomes smaller than the mechanical energy stored. This suggests that macroscopic mechanical energy can be used to very efficiently deform molecular machines in interfacial systems.

**Fig. 32:**
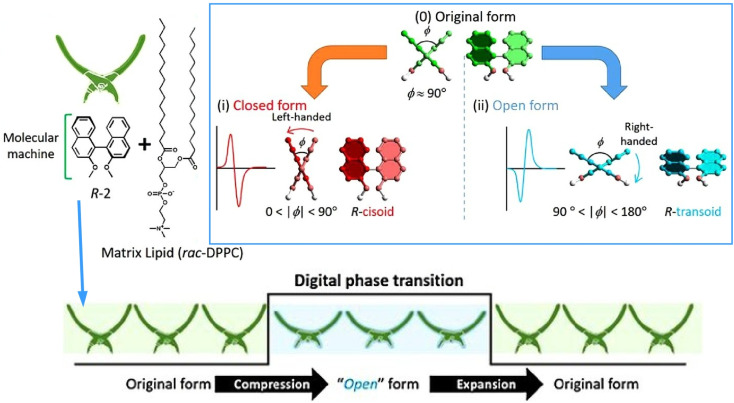
Binaphthyl derivatives, previously used as molecular pliers because they natively exhibit analogue transformation behaviour induced by monolayer compression, are converted to exhibit digital behaviour. This is accomplished by placing the binaphthyl derivative in a monolayer matrix, exploiting its phase transitions induced by mechanical stress to activate reversible cisoid–transoid conformational transitions (*i.e.* reversal of left- and right-handed helical structures), resulting in digital opening and closing of the molecular pliers. Reprinted with permission from ref. 63, Copyright 2017 Wiley-VCH.

Treating molecular machines as assemblies enables modulation of their behaviour. The open/closed state of the molecular pliers described above is an analogue transformation induced by monolayer compression. Alternatively, discontinuous, digital behaviour can be induced in the molecular pliers by placing them in a monolayer matrix and exploiting its phase changes. In the mixed monolayer system shown in Fig. 32, phase transitions induced by mechanical stress enable the digital opening and closing of simple binaphthyl molecular pliers.^63^ By applying mechanical force at the air–water interface, phase transitions in the monolayer matrix between a homogeneous liquid state and a metastable nanocrystalline state lead to reversible cisoid–transoid conformational transitions (*i.e.*, reversal of left- and right-handed helical structures) of binaphthyl derivatives. Since the conformational change is reversible, repeated crystallization and dissolution of metastable binaphthyl crystals induces repeatable alteration of the binaphthyl assembly's molecular structure. Thus, digital opening and closing of the molecular pliers can be realized through external mechanical stimulation at the air–water interface.

**Fig. 33:**
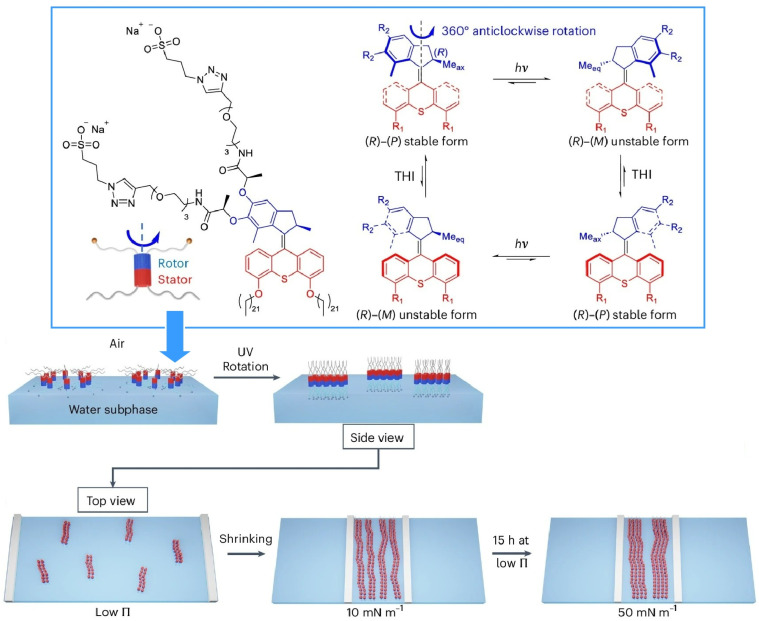
Supramolecular polymerization at the air–water interface induced by photo-stimulated rotation of molecular motors; the mechanical work generated by the rotation of individual motors drives the formation and growth of nanostructures. Reprinted with permission from ref. 64, Copyright 2025 Springer-Nature.

The air–water interface is an ideal medium for controlling the assembly of molecules and the clustering of molecular machines. Maaloum, Semenov, Giuseppone and co-workers reported supramolecular polymerization at the air–water interface induced by the rotation of molecular motors (Fig. 33).^64^ Providing the system with an appropriate energy source, such as photons, enables the autonomous, non-equilibrium motion of the monomers to be manipulated, thereby controlling the polymer structure and altering its growth dynamics. On average, the motors maintain a stable state, and the mechanical work generated by the photo-stimulated rotation of individual motors drives the formation and growth of nanostructures. This clustering phenomenon, which is controlled by precise and rapid nanomechanical motion, is conceptually distinct from thermal annealing processes. Unlike non-specific heating processes, the motion is confined to the driving parts of the molecular motors. This approach could lead to new ways of controlling soft matter at the nanoscale.

Macroscopic mechanical actions and molecular motions can be coupled at the air–water interface. Integrating molecular machines at this interface enables them to be driven by macroscopic mechanical stimuli and optimizes the function of molecular receptors. Furthermore, the efficiency of macroscopic energy consumption to drive molecular machines is extremely high. Despite its soft structure, the monolayer at the air–water interface secures free space that ensures the collective motion of molecular rotors and other molecules. At the air–water interface, collective properties, such as phase separation and supramolecular polymerization, can be adapted to drive the functions of molecular machines. The strategy of using molecular machines at liquid interfaces, most notably the air–water interface, has great potential to open up new scientific frontiers.

### Collective behaviour of biomolecular machines

Biological systems exhibit far more sophisticated functions than artificial supramolecular systems. They are home to numerous biomolecular machines, the most sophisticated and functional of which are proteins. Harvesting these biomolecular machines and artificially organizing them to function collectively is a promising approach for developing advanced functional systems. Some examples utilizing this strategy are introduced in the following sections.

**Fig. 34:**
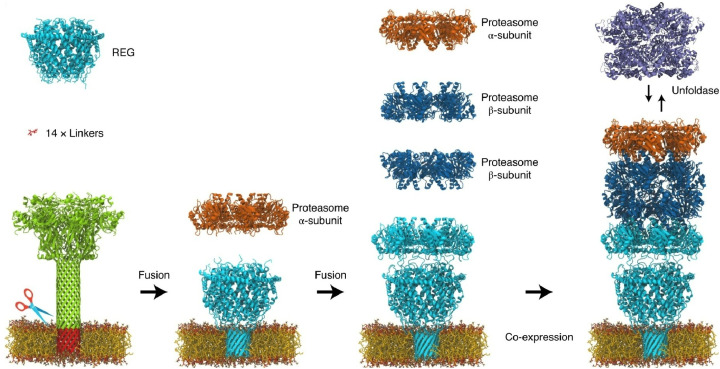
An integrated multiprotein complex that controls the unfolding and transport of individual proteins through a nanopore. Reprinted with permission from ref. 65, Copyright 2021 Springer-Nature.

The first example illustrates a method for creating nanopores with sophisticated machine capabilities *via* protein assembly. Maglia *et al.* engineered an integrated multiprotein complex that controls the unfolding and transport of individual proteins through a nanopore (Fig. 34).^65^ Replacing protein surface loops with the membrane-spanning region of a β-barrel pore, flanked by short hydrophilic linkers, enables cyclic, soluble proteins to be inserted into lipid membranes. Using this methodology, the archaeal 20S proteasome was incorporated into an artificial nanopore to control the unfolding and linear transport of proteins through the nanopore. Selected substrate proteins are unfolded and delivered to the proteasome chamber for processing into fragmented peptides or intact polypeptides. This enables new approaches for analysing proteins at the single-molecule level, à la protein sequencing. The nanopore could function as a stable, low-noise β-barrel nanopore sensor.

**Fig. 35:**
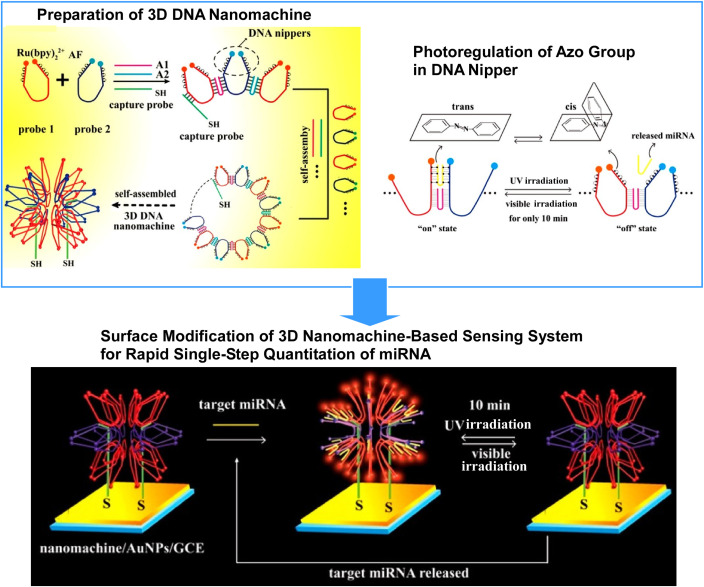
A methodology for the self-assembly of highly ordered, electric-field-free 3D DNA nanostructures using azobenzene-functionalized DNA nippers. The high local concentration of nippers organized on homogeneous DNA nanostructures enables them to function as 3D DNA nanomachines, boasting improved translocation efficiency over conventional 3D nanomachines. Reprinted with permission from ref. 66, Copyright 2018 American Chemical Society.

Yuan, Zhuo, Chai and co-workers demonstrated a methodology for the self-assembly of highly ordered, electric-field-free 3D DNA nanostructures using azobenzene-functionalized DNA nippers (Fig. 35).^66^ The high local concentration of nippers organized on homogeneous DNA nanostructures enables them to function as 3D DNA nanomachines with improved translocation efficiency over conventional ones. Incorporating azobenzene moieties into the DNA nippers preserves the nanostructure of the DNA nanomachine and enables the reversal of nanomachine translocation in a single, rapid step. In the presence of a target microRNA (miRNA), hybridization between the miRNA and the nipper generates an open state, reflected by an enhanced electrochemiluminescence (ECL) signal from Ru(bpy)_2_^2+^ due to its proximity to the Alexa Fluor quencher. This method can be applied in rapid, single-step detection of biomarkers, with applications in sensing, analysis, and diagnostics. Cancer-cell-derived biomarkers can be analysed quantitatively with ultra-high sensitivity in a rapid single step process. This could provide a route to next-generation nanomachines for early cancer detection and diagnosis.

**Fig. 36:**
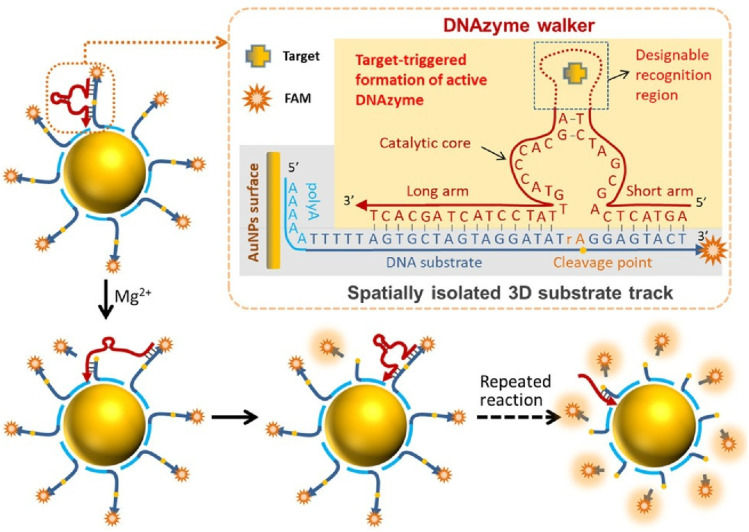
Spatially separated 3D DNA tracks adsorbed onto AuNPs *via* polyadenine (polyA), and a target-initiated DNAzyme walker; together the molecules form a sensor that sensitively and selectively detects adenosine, Ag^+^, and target DNA. Reprinted with permission from ref. 67, Copyright 2018 American Chemical Society.

Collectively operating synthetic DNA machines on nanoscale 3D tracks are rapidly gaining attention due to their potential applications in areas such as biocomputing, drug delivery and biosensing. Yang, He and co-workers were the first to report spatially separated 3D DNA tracks based on polyadenine (polyA) (Fig. 36).^67^ They developed a walking device consisting of a target-activated DNAzyme walker and the 3D track adsorbed onto an AuNP surface *via* polyA. The DNAzyme walker moves along this track *via* a burnt-bridge mechanism, through which it demonstrates improved efficiency and throughput. The DNAzyme-walking-device-based sensor demonstrated cascade signal amplification resulting in selective and sensitive detection of adenosine, Ag^+^, and target DNA. This demonstrates great potential in high-performance, bioinspired DNA nanomachines.

**Fig. 37:**
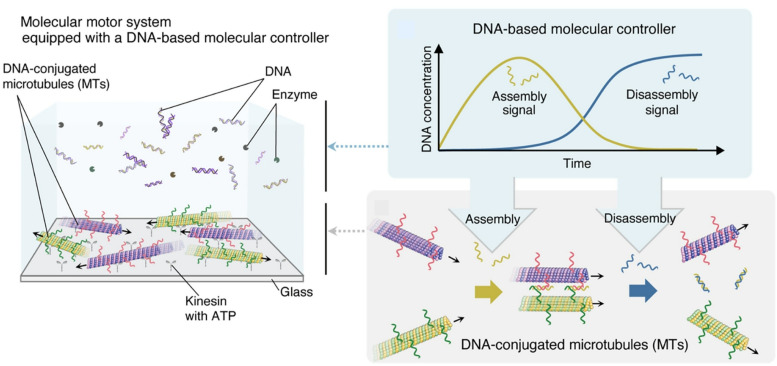
A DNA-based cascade reaction functioning as a molecular controller, driving the autonomous assembly and disassembly of DNA-functionalized microtubules *via* kinesin. The system comprises three DNA complexes and three enzymes to autonomously execute each step of the cascade, in the absence of any external stimuli. Reproduced under the terms of the CC-BY license from ref. 68, 2024 American Association for the Advancement of Science.

Current systems often rely on manual application of external stimuli, which limits the potential of autonomous molecular systems. In a breakthrough, Kakugo, Nomura and co-workers demonstrated that a DNA-based cascade reaction can function as a molecular controller, driving the autonomous assembly and disassembly of DNA-functionalized microtubules *via* kinesin (Fig. 37).^68^ The system comprises three DNA complexes and three enzymes, referred to as the template, converter, and transducer. The three enzymes, a polymerase, nickase and restriction enzyme, can synthesize new DNA from template DNA, cleave half of a double-stranded DNA and completely cleave double-stranded DNA, respectively. By designing a reaction cascade consisting of cDNA hybridization, DNA strand displacement, and enzyme activity, the molecular controller can autonomously execute each step of the cascade. Gliding microtubules integrated into the controller assemble into bundled structures and disassemble into individual filaments in the absence of any external stimuli. This introduces a new concept of autonomously controlled materials driven by molecules and equipped with a smart controller that encodes the system's instructions.

**Fig. 38:**
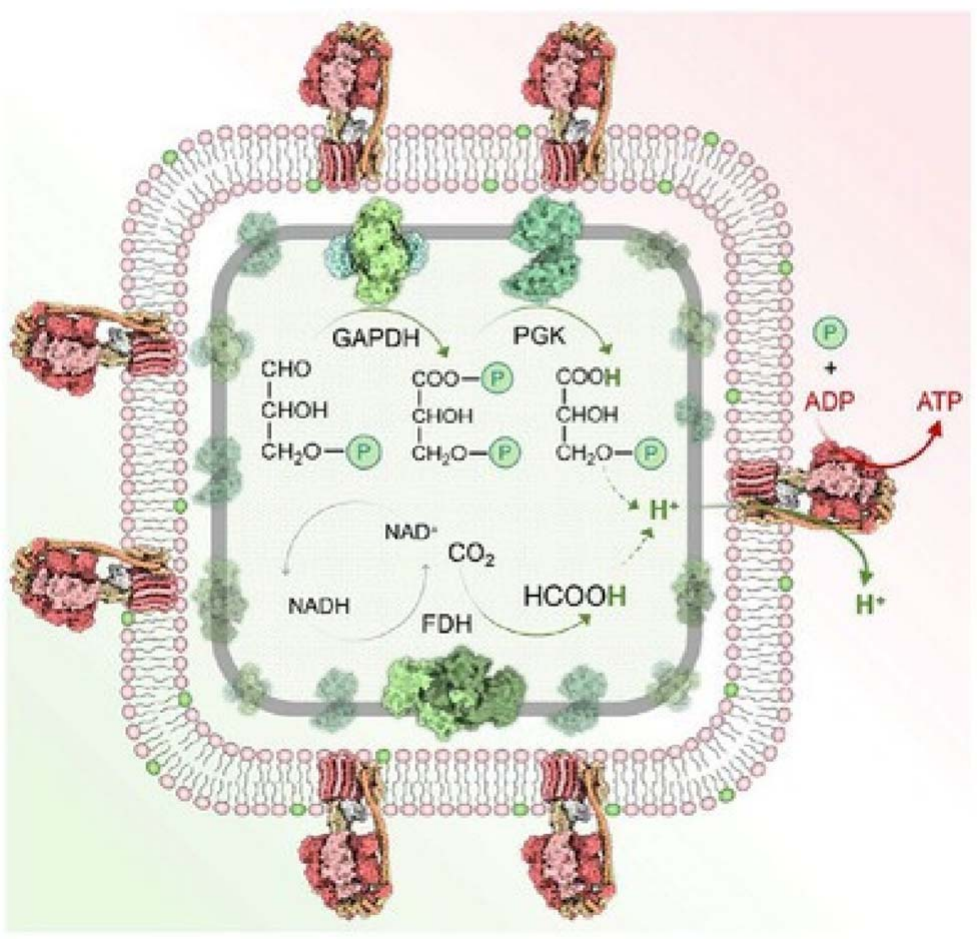
Co-immobilization of glyceraldehyde-3-phosphate dehydrogenase (GAPDH), 3-phosphoglycerate kinase (PGK), formate dehydrogenase (FDH) and ATPase-incorporated proteoliposomes on hollow polydopamine (HPDA), which serves as a biocompatible scaffold. This creates a microreactor, wherein GAPDH and PGK catalyse the conversion of glyceraldehyde-3-phosphate to 3-phosphoglycerate, reducing β-nicotinamide adenine dinucleotide (NAD^+^) to NADH and generating a proton influx to drive ATP synthesis. The microreactor has a strong affinity for CO_2_, a property exploited with the aid of FDH to reduce CO_2_ to formate and oxidize NADH to NAD^+^, recycling the NAD^+^/NADH redox couple for efficient energy synthesis from low-value substrates. Reprinted with permission from ref. 69, Copyright 2025 Wiley-VCH.

Jia *et al.* reported a system in which glyceraldehyde-3-phosphate dehydrogenase (GAPDH), 3-phosphoglycerate kinase (PGK), formate dehydrogenase (FDH) and ATPase-incorporated proteoliposomes were co-immobilized using hollow polydopamine (HPDA) as a biocompatible scaffold (Fig. 38).^69^ Within this microreactor, GAPDH and PGK catalyse the conversion of glyceraldehyde-3-phosphate to 3-phosphoglycerate, reducing β-nicotinamide adenine dinucleotide (NAD^+^) to NADH and generating a proton influx to drive ATP synthesis. The microreactor exhibits a strong affinity for CO_2_, a property exploited with the aid of FDH to facilitate the reduction of CO_2_ to formate and the oxidation of NADH to NAD^+^, enabling recycling of the NAD^+^/NADH redox couple. This process also supplies protons to drive ATP production. This nanoarchitectonic microreactor expertly coordinates both chloroplast and mitochondrial enzymes, essentially recreating the functional coordination of these organelles *in vitro*. We anticipate that this approach will inspire methods for more efficient ATP synthesis from low-value substrates, with potential to address environmental and energy challenges.

**Fig. 39:**
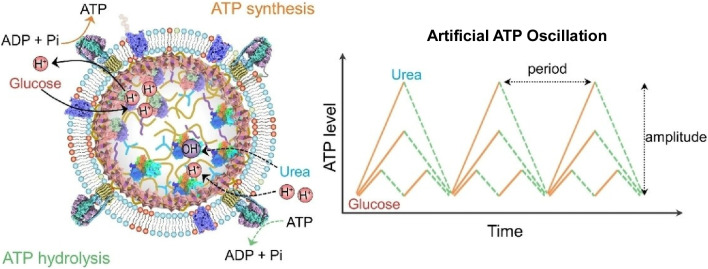
A compartmentalized nanostructure used to regulate bioenergetic levels. Encapsulated glucose dehydrogenase (GDH), urease and nicotinamide adenine dinucleotide drive an oscillatory proton gradient, which creates an oscillatory cycle of ATP synthesis and decomposition controlling bioenergetic levels in opposing directions. Reprinted with permission from ref. 70, Copyright 2024 Wiley-VCH.

Fei, Li and co-workers constructed a compartmentalized nanostructure to regulate bioenergetic levels (Fig. 39).^70^ In the assembled structure, glucose dehydrogenase (GDH), urease and nicotinamide adenine dinucleotide were encapsulated by liquid–liquid phase separation. ATPase and glucose transporters embedded in hybrid liposomes were attached to the capsule surface. In this nanostructure, glucose is transported and converted to gluconate by GDH, creating an outward proton gradient to drive ATP synthesis. In parallel, urease catalysed the hydrolysis of urea to produce ammonia, creating an inward proton gradient to drive ATPase for ATP decomposition. These processes alter the direction of the proton gradient, resulting in artificial ATP oscillations. The frequency and amplitude of these oscillations can be precisely tuned by varying the loading of the encapsulated enzymes and the fuel concentrations (glucose and urea) within the nanostructure. This design innovates a new strategy for developing nanoarchitectures that can control bioenergetics levels in opposing directions.

The systems described above highlight how multiple types of biomolecular machines can work in concert. These biomolecular machines are not limited to collective action of singular molecular species; there are also systems of diverse biomolecular machines collaborating. A major advantage of biomolecular machine assemblies is that they excel at coordination of diverse molecular machines serving different functions within the system, creating a flow of function. Biomolecular machines have sophisticated and highly functional capabilities, and their coordinated action can yield functions rivalling or even surpassing those of living organisms. Developing host materials to integrate these biomolecular machines, in addition to nanoarchitectonics approaches for their manipulation, is of the utmost importance.

### Collective behaviour of microrobots

In addition to molecular and biomolecular machines, there are also nanorobots and microrobots: nano- and microscale functional materials that behave like machines. Distinct from molecular machines, they are designed with and synthesized from nanomaterials and micromaterials rather than individual molecules or biomolecules. Although they are larger than molecular machines, their collective behaviour is no less interesting or functional. In this section, we examine several examples of studies featuring collective behaviour of microrobots.

**Fig. 40:**
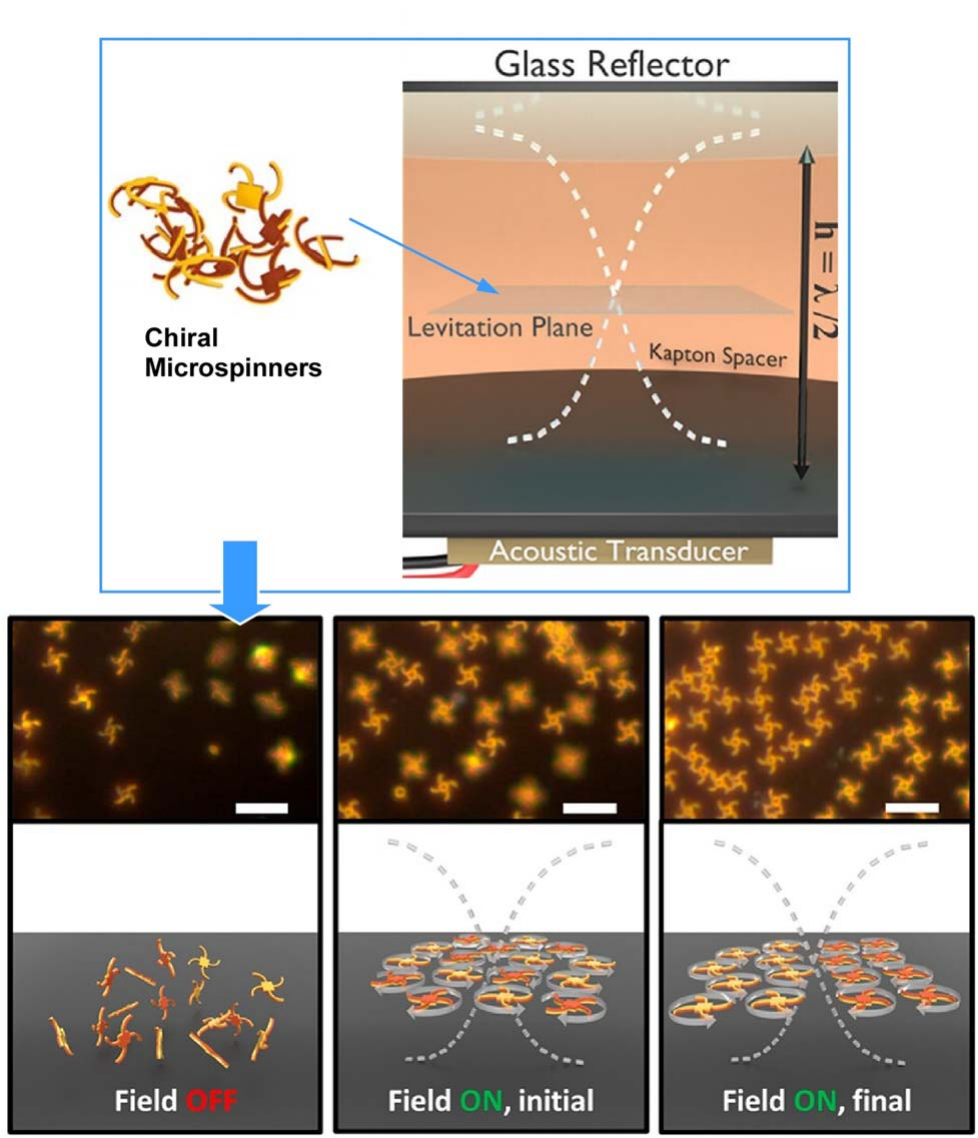
Switchable, non-equilibrium, hydrodynamic assembly and phase separation in a suspension of acoustically driven, chiral micro-spinners. The 3D chirality of the spinners drives self-assembly in parallel planes, forming a 3D hierarchical system. The twisted 3D structure introduces system-wide chirality. Reprinted with permission from ref. 71, Copyright 2023 American Chemical Society.

Controlling the interactions of large-scale synthetic particle swarms could lead to similar macroscopic robotic systems possessing microscopic complexity. Rotation-induced self-assembly has been reported in biological systems. Kagan, Mallouk and co-workers reported switchable, non-equilibrium, hydrodynamic assembly and phase separation in a suspension of acoustically driven, chiral micro-spinners (Fig. 40).^72^ Semiquantitative modelling suggests that in this system, the 3D complex spinners interact *via* viscous forces and weak inertial streaming. The 3D chirality of the spinners drives self-assembly in parallel planes, forming a 3D hierarchical system. This twisted 3D structure effectively introduces system-wide chirality. Such 3D microspinners may provide a framework for engineering more complex systems at the particle level, with anticipated contributions in physical science. Further effort towards incorporating new layers and complex particle types is anticipated.

**Fig. 41:**
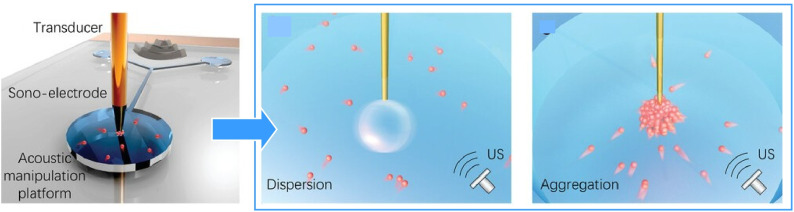
Dispersing and aggregating individual micromotors using a needle-like hybrid sonoelectrode. Hydrogen bubbles are generated at the tip of a charged electrode and vibrated by an acoustic field to create strong vortices that disperse the micromotors spontaneously. By removing the bubbles, the sonoelectrode acts as a solid-needle isolator that drives micromotor aggregation. Reprinted with permission from ref. 72, Copyright 2021 Wiley-VCH.

Controlled release and precise enrichment of microrobot swarms have the potential to significantly enhance detection speeds and selectivity in biosensing and chemical analysis. Lu *et al.* demonstrated an efficient and versatile strategy for dispersing and aggregating individual micromotors using a needle-like hybrid sonoelectrode (Fig. 41).^72^ This strategy significantly accelerates mass transfer and enhances the signal intensity. In their system, hydrogen bubbles are generated at the tip of a charged electrode and vibrated by an acoustic field to create strong vortices that disperse the micromotors spontaneously. By removing the attached bubble, the sonoelectrode acts as a solid-needle isolator. This generates acoustic streaming within the actuation reservoir using high-frequency ultrasound, enabling the large-scale collection of micromotors. This system has the potential to control micromotor swarming behaviour with versatility and speed, facilitating the development of intelligent microrobots with active enrichment capabilities compatible with next-generation, highly sensitive, portable detection microsystems. This strategy could be applied in next-generation wearable microsystems for active, high-precision biochemical analysis and ultratrace-level detection.

**Fig. 42:**
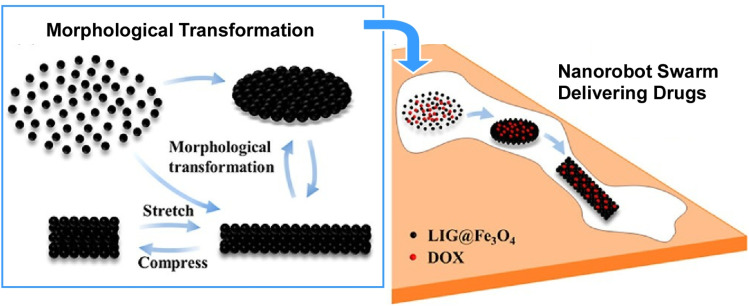
Laser-induced graphene (LIG)-based Fe_3_O_4_ nanorobot swarms form various reversible morphologies, including vortices and strips in response to magnetic fields. Drug-loaded swarms can pass smoothly through zigzag channels of varying widths while retaining 96% of the initial drug loading for targeted release. Reprinted with permission from ref. 73, Copyright 2024 American Chemical Society.

Magnetic nanorobot swarms can mimic collective behaviour found in nature. These swarms can be flexibly controlled with programmable magnetic fields, boasting great potential for a variety of applications. Chen *et al.* reported a novel approach to the rapid and scalable fabrication of laser-induced graphene (LIG)-based Fe_3_O_4_ nanorobot swarms using a one-step UV laser processing technique (Fig. 42).^73^ These swarms can form various reversible morphologies, including vortices and strips, in response to magnetic fields. Furthermore, these morphologies can interconvert, demonstrating high controllability and flexibility. The drug loading and release performance utilizing these nanorobot swarms improved by approximately 50-fold compared to individual carriers. Furthermore, drug-loaded swarms can pass smoothly through zigzag channels of varying widths while retaining 96% of the initial drug payload. Loading, release, and targeting of doxorubicin (DOX) using these nanoparticle swarms under magnetic field control has been demonstrated. This study sheds light on the processing of nanorobot swarms and their application as high-performance, targeted drug delivery systems.

**Fig. 43:**
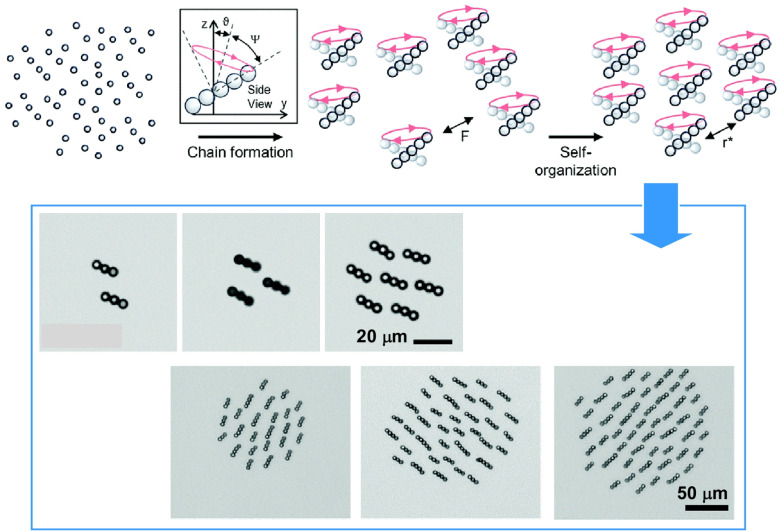
Paramagnetic particles dynamically self-organize of pinto anisotropic linear chains. These linear chains act as microrobots that further self-organize into clusters when they spontaneously align under induced magnetic moments. As the clusters increase in size, they transition from a solid-like state to a liquid-like state. Additionally, cluster velocity also increases with size due to collective hydrodynamic effects. Reproduced under the terms of the CC-BY license from ref. 74, 2020 Royal Society of Chemistry.

Many high demand and high impact applications require mobile microrobots to form cohesive swarms in unbounded conditions. Sitti and his team discovered that balancing magnetic dipole attractions and multipole repulsions enables self-assembled particle-chain microrobots to self-organize into cohesive clusters (Fig. 43).^74^ The microrobots themselves were formed through dynamic self-organization of paramagnetic particles into anisotropic linear chains. At a specific opening angle of the processing magnetic field, magnetic interactions between the chains induce slowly decaying dipole attractions and rapidly decaying multipole repulsions. These opposing effects result in an equilibrium distance between pairs where the sum of the dipole and multipole forces is zero. Under cohesive interactions, the chains self-organize into clusters by aligning at an equilibrium distance from neighbouring chains. The scalability of cohesive interactions enables larger groups to form, and their internal spatiotemporal organization can transition from a solid-like state to a liquid-like state as the cluster increases in size. Additionally, cluster velocity was found to increase with cluster size due to collective hydrodynamic effects. This work achieved operation of microrobots as localized swarms, and has potential to inspire the design of advanced ensemble systems for application in biomedicine, precision manipulation and manufacturing, and environmental sensing and remediation.

Many biological systems, such as birds and fish, exhibit collective behaviours such as schooling. Analogous behaviour has been reported in active colloidal particles, which coordinate the movement of subsidiary particles that make up microrobots. Liu and Dijkstra used Brownian dynamics simulations to study the 3D collective behaviour of intelligent active Brownian spheres, rods and nematodes.^75^ This study used computer simulations to examine intelligent active Brownian particles with visual perception and 3D velocity alignment. These particles exhibit a tendency to move towards their centre of mass while velocity alignment promotes synchronization with neighbouring particles. Reducing the size of the simulation box revealed new structures, such as band-like clusters and dense “baitballs”. Rod-like particles formed band-like, worm-like, radial and spiral structures. Worm-like particles exhibited band-like, streamlined, micellar and entangled structures. Many of these patterns resemble collective behaviours observed in nature, such as ant milling, fish baitballs and worm-like clusters. Advances in synthesis could lead to the creation of nanorobots with similar functions, which could in turn provide valuable insights into multicellular systems by way of studying active substances.

**Fig. 44:**
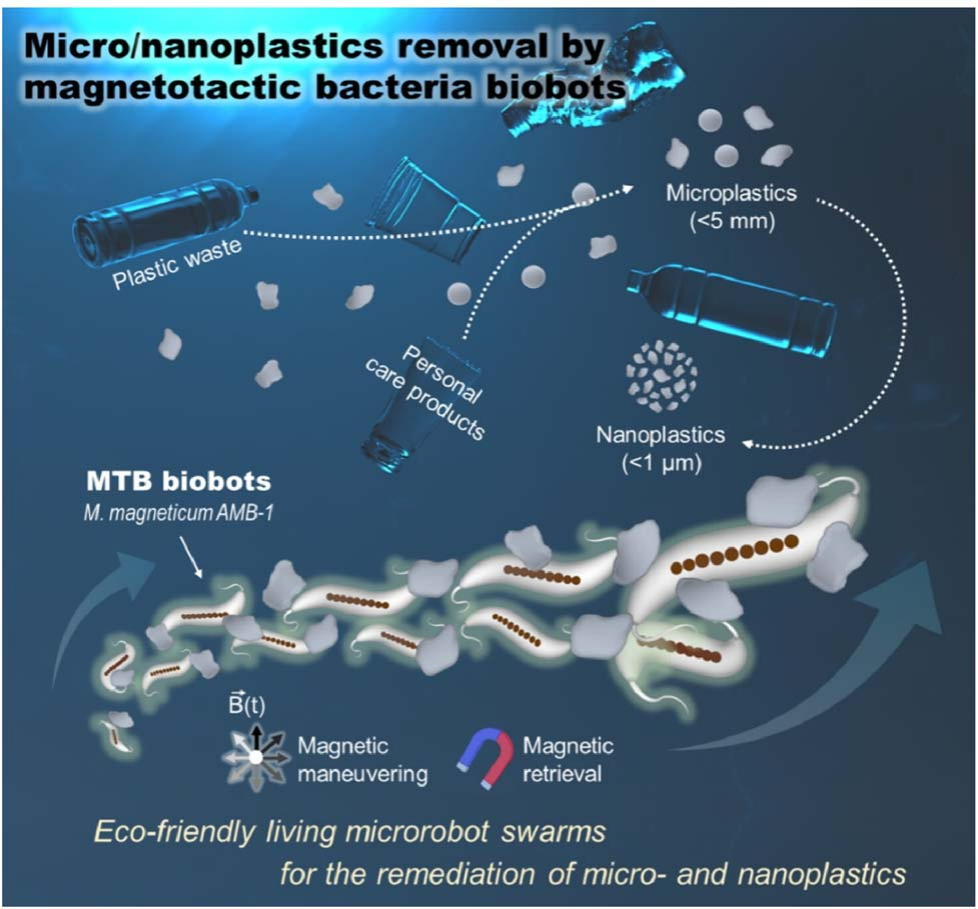
Magnetically driven swarm of living bacterial microrobots for the purification of aquatic microplastics and nanoplastics. The microrobots can propel themselves *via* flagellar movement. Additionally, they can be collectively steered with a rotating magnetic field. The multimodal operation of these microrobots which replicates 3D swarming navigation, not unlike a school of fish. This allows active capture of micro- and nanoplastics in a biomechanical solution to the microplastics pollution crisis. Reproduced under terms of the CC-BY license from ref. 76, 2025 American Chemical Society.

As mentioned above, the collective behaviour of microrobots exhibits similarities to those seen in biological systems. Some researchers have built on this concept by incorporating living organisms into microrobots. For example, Pumera and co-workers proposed a magnetically driven swarm of living bacterial microrobots for the purification of aquatic microplastics and nanoplastics (Fig. 44).^76^ The combination of magnetically guided navigation and autonomous propulsion enables the multimodal operation of a swarm of magnetotactic-bacteria-based living microrobots. A rotating magnetic field induces 3D swarming navigation similar to that of a school of fish, actively capturing micro- and nanoplastics. These are then recovered from contaminated water by magnetic separation. Actuation of these microrobots combines autonomous self-propulsion *via* flagellar movement with precise, untethered navigation controlled *via* the magnetic field. This enables multimodal steering, such as directional propulsion or rotary circular motion. These magnetotactic-bacteria-based living microrobots exhibiting swarm behaviour present a biomechanical solution that may find use in the impending microplastics pollution crisis.

Although microrobots are larger than molecular machines, they share the same reliance on collective structural organization. Their behaviour is not unlike a swarm of living organisms, and their use in practical applications such as the cleanup of microplastics shows promise. Their advantage over molecular machines is ease of observation and simulation. The collective behaviour of microrobots is very important, as the collective action of many units sum ups to larger-scale function. While the effects of microscopic thermal fluctuations and Brownian motion on molecular machines and microrobots differ, microrobot behaviours could nevertheless yield valuable insights into the collective behaviour of molecular machines.

## Future perspective

This review has explored collective behaviours in molecular machines and microrobots, finding trends in collective behaviours and their associated material systems and environments. We have placed emphasis on the paradigm shift from solitary to collective behaviour, examining how this is reflected in functionally organized materials. Applying the concept of nanoarchitectonics to molecular machines and microrobots could facilitate the deliberate design of functional material systems at the nanoscale.

We identified several distinct collective behaviour patterns, the most notable of which are: (i) multiple molecular machines operating independently, with the sum of unique molecular functions producing a significant macroscopic output; (ii) conversely, collective structures can also drive individual molecular machines through macroscopic actions and forces; (iii) more advanced collective structures in which molecular machines work synchronously like force-transducing gears; and (iv) even without physical, gear-like force transduction interactions, systems of diverse molecular machines (including biomolecular machines) with linked outputs can produce more advanced mechanical and functional system outputs.

The many examples presented in this review demonstrate that the collective behaviours of molecular machines are strongly correlated with their environment. This can be summarized as follows: (i) regularly ordered porous materials, such as metal–organic frameworks (MOFs) and covalent organic frameworks (COFs), provide nanospaces in which molecular machines can move freely. The collective action of molecular machines within MOFs and COFs is an attractive strategy for converting molecular function into practical output. (ii) Crystals, despite not providing nanospaces like MOFs do, instead provide ordered structures in which molecular machines can perform collective behaviours in a controlled manner. The ability to construct materials with integrated molecular gear structures provides a methodology for coupling motion at the molecular level with macroscopic material properties. Furthermore, the shape and motion of molecular machines can be directly observed on solid surfaces, enabling the design of sophisticated collective behaviours involving molecular gears meshing and interacting with molecular rotors. The operational behaviour of molecular machines can also be studied at the quantum level through molecular observations using STM and theoretical calculations. (iii) At the air–water interface, coupling of macroscopic mechanical motion to molecular motion can be achieved. Integrating molecular machines at an interface enables them to be driven by macroscopic mechanical stimuli and allows the function of molecular receptors to be optimized. Using liquid interfaces such as the air–water interface is a methodology with great potential for opening up new scientific fields.

Expanding the scope from relatively small, organically synthesized molecular machines to biomolecular machines made from large biological molecules or even larger microrobots, different collective characteristics are observed. One major advantage of systems in which multiple biomolecular machines work together is their suitability for creating function flows integrating the effects of different machines in a single system. Biomolecular machines have innately sophisticated functions, and their organizational manipulation can yield larger functions greater than the sum of their parts. This is not unlike collective behaviours in living organisms, and artificial, deliberate design is anticipated to give form to machines comparable to, or even surpassing those seen in living organisms. These characteristics have practical importance, as their application to solving real world problems such as the recovery of microplastics has been demonstrated.

Molecular machines are at the pinnacle of nanoscience, having realized nano- and microscopic objects that function like machines. They have even won a Nobel Prize. However, more technological advancements are necessary before these materials find widespread practical use. A crucial step is to assemble individual molecular machines into objects that can function collectively, with the aid of nanoarchitectonics methodologies. This review found correlations between molecular machines' properties and their environment and morphology, identifying tendencies for certain collective forms to exhibit correspondent functions. Interfaces are particularly enabling for organizational manipulation of molecular machines to result in useful and impactful functions. To advance the practical application of these sciences and technologies, holistic combination of conventionally distinct research fields is necessary to inspire designs transcending the systems described in this work. In particular, the deliberate design and synthesis of functional systems combining and coordinating the functions of multiple molecular machine elements is essential. Designing such complex systems requires synthesis informed by accumulated experience from countless materials science experiments and vast amounts of data. As such, the intervention of artificial intelligence (AI), and moreover AI-based nanoarchitectonics, has been in demand.^77^ Building complex assemblies by manipulating molecular machines using this approach is anticipated to accelerate the fruition of highly functional systems that match, or even exceed the strategies used by the living organisms that inspired them. This is an ultimate goal for functional materials chemistry.

## Author contributions

Conceptualisation K. A. and J. S.; collecting literature K. A., W. L. and J. S.; writing – original draft, review and editing K. A., W. L. and J. S.; supervision K. A.; funding acquisition K. A.

## Conflicts of interest

There are no conflicts to declare.

## Data Availability

No primary research results, software or code have been included, and no new data were generated or analysed as part of this review.
